# When does the use of individual patient data in network meta-analysis make a difference? A simulation study

**DOI:** 10.1186/s12874-020-01198-2

**Published:** 2021-01-13

**Authors:** Steve Kanters, Mohammad Ehsanul Karim, Kristian Thorlund, Aslam Anis, Nick Bansback

**Affiliations:** 1 School of Population and Public Health, University of British Columbia, 2206 E Mall, Vancouver, British Columbia Canada; 2 Centre for Health Evaluation and Outcome Science, University of British Columbia, Vancouver, Canada; 3 Departments of Health Research Methods, Evidence & Impact, McMaster University, Hamilton, Canada

**Keywords:** Individual patient data, IPD, Network meta-analyses, NMA, Simulation study, Methods

## Abstract

**Background:**

The use of individual patient data (IPD) in network meta-analyses (NMA) is rapidly growing. This study aimed to determine, through simulations, the impact of select factors on the validity and precision of NMA estimates when combining IPD and aggregate data (AgD) relative to using AgD only.

**Methods:**

Three analysis strategies were compared via simulations: 1) AgD NMA without adjustments (AgD-NMA); 2) AgD NMA with meta-regression (AgD-NMA-MR); and 3) IPD-AgD NMA with meta-regression (IPD-NMA). We compared 108 parameter permutations: number of network nodes (3, 5 or 10); proportion of treatment comparisons informed by IPD (low, medium or high); equal size trials (2-armed with 200 patients per arm) or larger IPD trials (500 patients per arm); sparse or well-populated networks; and type of effect-modification (none, constant across treatment comparisons, or exchangeable). Data were generated over 200 simulations for each combination of parameters, each using linear regression with Normal distributions. To assess model performance and estimate validity, the mean squared error (MSE) and bias of treatment-effect and covariate estimates were collected. Standard errors (SE) and percentiles were used to compare estimate precision.

**Results:**

Overall, IPD-NMA performed best in terms of validity and precision. The median MSE was lower in the IPD-NMA in 88 of 108 scenarios (similar results otherwise). On average, the IPD-NMA median MSE was 0.54 times the median using AgD-NMA-MR. Similarly, the SEs of the IPD-NMA treatment-effect estimates were 1/5 the size of AgD-NMA-MR SEs. The magnitude of superior validity and precision of using IPD-NMA varied across scenarios and was associated with the amount of IPD. Using IPD in small or sparse networks consistently led to improved validity and precision; however, in large/dense networks IPD tended to have negligible impact if too few IPD were included. Similar results also apply to the meta-regression coefficient estimates.

**Conclusions:**

Our simulation study suggests that the use of IPD in NMA will considerably improve the validity and precision of estimates of treatment effect and regression coefficients in the most NMA IPD data-scenarios. However, IPD may not add meaningful validity and precision to NMAs of large and dense treatment networks when negligible IPD are used.

**Supplementary Information:**

The online version contains supplementary material available at 10.1186/s12874-020-01198-2.

## Background

The use of network meta-analysis (NMA) has grown exponentially over the past few years [1]. With its increased use has come a number of methodological developments, including the expansion from aggregate data (AgD) to the combined use of individual patient data (IPD) and AgD [2]. Many of these newer methods have been highlighted in the recent, and highly influential, National Institute for Health Care and Excellence (NICE) Technical Support Document 18 [3]. Chief among them are population-adjusted indirect comparisons (PAIC), such as matched indirect comparisons (MAIC) [3]. The NICE guidance demonstrates that PAICs can be used in connected networks to adjust for imbalances in effect-modifiers and in disconnected networks to adjust for both effect-modifiers and other prognostic factors. While both are feasible, the guidance emphasizes that PAIC is more ideally used in connected networks where the aim is to make better adjustments of imbalances in effect-modifiers only. The difference is akin to using randomized trials for causal inference and propensity score-adjusted analyses of observational studies. A great achievement of PAIC has been its important uptake, both in the general research community [4] and within the health technology assessment (HTA) community [5]. The latter has been particularly important in the current pharmaceutical climate that often sees treatments fast-tracked through development due to very promising early results, which can lead to non-comparative studies. While the first phase of uptake of IPD use within HTA submissions has been principally focused on disconnected networks, a consistent criticism of such analyses has been the lack of prognostic factors being adjusted for [5].

NICE guidance acknowledges that there are numerous ways to combine IPD and AgD, in addition to PAIC. These methods are restricted to connected networks, thus avoiding the criticism regarding the need for prognostic factors. Primarily, they include: a two-stage approach where data are first transformed into AgD only and then analyzed traditionally; a one-stage approach where IPD and AgD are analyzed simultaneously, and other more under-developed methods, such as hierarchical meta-regression, which may reduce some forms of bias. Despite the acknowledgement of these other methods, little guidance is provided for these methods; in part due to lack of evidence surrounding their performance. Thus, while the uptake of PAIC is partially due to its ability to handle disconnected networks, it is also due to the clearer guidance that has been provided for these methods. It is anticipated that as better understandings of other IPD-AgD methods become available, and that in turn better guidance is provided, the uptake of other methods will increase substantially.

Effect modification occurs in NMA, when one or more variables that impact the treatment-effect, dubbed effect modifiers, are imbalanced across different edges of the network. In order to move forward with better adjustments for imbalances in effect-modifiers in networks of evidence, the one-stage approach seems destined to play a larger role in evidence synthesis moving forward. The reason is two-fold. First, relative to the two-stage approach, a one-stage approach takes full advantage of the data in a single analysis rather than adjusting each IPD trial separately. Second, it allows for adjustments in larger networks than using MAIC, which can only make adjustments in small networks (3 nodes at a time).

Empirically, the use of IPD in NMA has generally been seen to have a large impact on their results; however, that isn’t always the case [2, 4, 6]. There are a number of factors that could explain why IPD adjustments can have minimal impact on the evidence synthesis results. Ideally, the underlying reason is due to the evidence base not being imbalanced with respect to effect-modifiers (i.e., because there are no adjustments needed to the data). An alternative may be that there are simply not enough IPD to make a meaningful impact. Donegan et al. conducted a one-stage NMA in which IPD made up 30% of patients and 25% of trials covering much of the network geometry, and found a meaningful impact [6]. In their discussion, the authors state: “*It would be interesting to compare the proposed approach with AD meta-analysis of all studies, while varying the number of studies that contribute IPD, to establish whether equally dramatic improvements are observed*.” [6]

In this study, which was part of a doctoral thesis [7], we aim to determine through the use of simulations, *‘how much IPD is enough to make a difference’.* Put in more absolute terms, we aim to examine if select factors are predictive of whether the use of IPD will lead to improvements in the validity and precision of estimates of comparative treatment-effects and meta-regression coefficients within NMA. In particular, the factors include factors specific to the individual patient data, namely the proportion of treatment comparisons and number of patients for which individual patient data are available; factors pertaining to the network of evidence, namely its number of nodes and whether it is sparsely populated; and the presence or absence of effect-modification and whether it was fully or partially shared across the comparisons in the network.

## Methods

We performed simulations of several AgD-IPD NMA data scenarios by varying the following data properties across scenarios: proportion of treatment comparisons with IPD, proportion of patients for which IPD are available, number of nodes in the network, network density, and the nature of effect-modification in the network [8]. To ensure that observed differences could be attributed to these parameters, each was varied individually and all other factors were kept constant. Consistently across all individual simulated scenarios, three NMA models were used to analyze the data: 1) AgD NMA without adjustments (AgD-NMA)); 2) AgD NMA with meta-regression (AgD-NMA-MR); and 3) IPD-AgD NMA with meta-regression (IPD-NMA).

### Simulation model parameters – outline and rationale

The simulation model parameters are described in Table 1. The values for the proportion of treatment comparisons and network density in the table were purposely broad because their meanings are codependent (see Data Generation below). Varying the number of nodes in the network (3, 5 and 10 nodes) was highly motivated by the desire to understand the impact of IPD in larger networks, which is not feasible using MAIC. The reason for including the trial sizes model parameter is to improve the differentiation between the number of patients available and the proportion of treatment comparisons with IPD, which is best captured through the proportion of treatment comparisons with IPD. Together, these factors are critical to answering the motivational question: How much IPD are needed to make a difference?

**Table 1:** List of the parameters explored through simulation with descriptions

Factor	Categories	Description and comments
Number of nodes (treatments) in the network	3	The number of nodes speaks directly to the principal objective of whether too few IPD data will have an affect the estimated treatment-effects in a noticeable manner. More nodes means more data are required for full coverage.
	5	
	10	
Proportion of treatment comparisons with IPD	Low	Low implied only a single treatment comparison with IPD. Medium implied multiple edges with IPD, but among the lower number of multiple edges possible for the given network. High allowed for up to 100% of edges having IPD
	Medium	
	High	
Effect-modification	None	The relationship between the covariate X and the relative treatment-effects. **None** indicates no relationship (treatment-effects are unchanged by varying values of the covariate). **Constant** indicates that the linear relationship between the covariate treatment-effect has the same slope for all treatment-effects relative to the reference treatment. **Exchangeable** indicates that the slope between covariate and treatment-effect changes according to the treatments being compared, but that they come from a common distribution of slopes. (see Fig. 1**)**
	Constant	
	Exchangeable	
Trial sizes	All trials of equal sizes	All trials had 200 patients per arm when set to equal. When IPD were larger, the IPD trials had 500 patients per arm. All trials were 2-arm trials.
	IPD trials are bigger	
Network density	Sparse	Sparse networks were star networks with no closed loops. They had 1–3 trials per treatment comparison. Well-populated trials had closed loops and treatment comparisons with up to 7 trials.
	Well-populated	

**Fig. 1:**
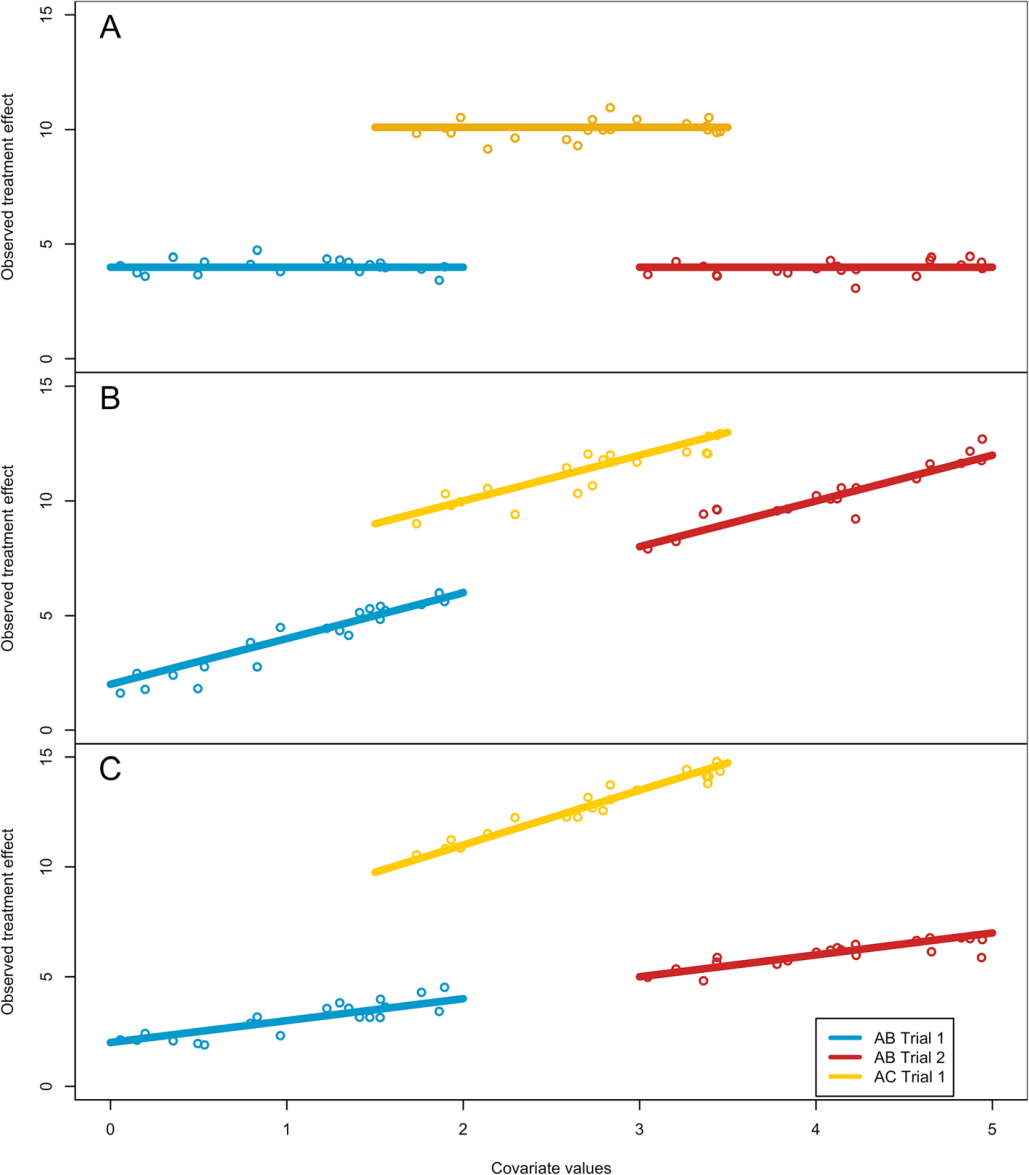
Illustration of the different types of effect-modification

The type of effect-modification is an intrinsic characteristic regarding the nature of the impact of the covariates on the treatment-effects. These would not be known to the researcher, except through clinical hypothesizing. Figure 1 presents the three types of effect-modification that were tested. Consistent effect-modification implies the relationship between outcome and covariate is shared between all trials. Exchangeable effect-modification implies the relationship is contrast-specific (shared by trials with the same comparison). While the slopes differ for each contrast, the slopes come from a distribution of slopes. Thus, in the exchangeable model there is a shared mean effect-modification from the distribution of possible effect-modifications.

### Data generation

**Fig. 2:**
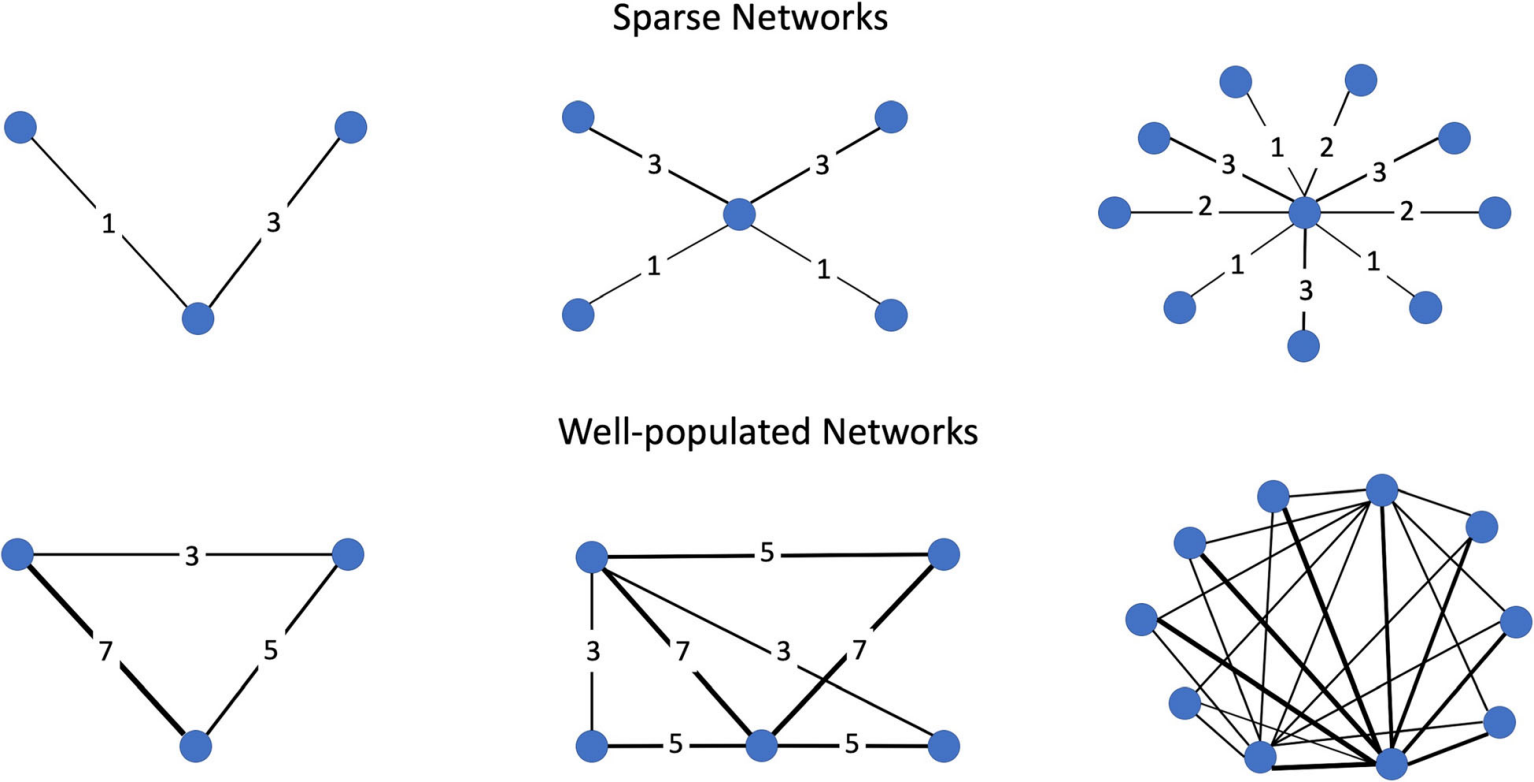
Illustration of the networks constructed using the number of nodes and network density

For simplicity, only two-arm trials were simulated. All trials included 200 patients per arm. If IPD trial sizes were set to large, then the trials selected as having IPD were set to having 500 patients per arm. The first step in creating the data was to determine the number of trials in the network (N) and the number of patients per trial arm (n_i_). Figure 2 depicts the number of trials used according to the combination of number of nodes and network density (see Web Appendix for precise counts). This step allowed the construction of the treatment (t) matrix for the AgD data to be built and hence construct empty y, se and n matrices to be filled in subsequently. The treatment matrix comprised of treatment numbers *t*_*jk*_ for each arm *k* of trial *j*.

The second step was to identify the IPD trials. The number of treatment comparisons with IPD was not fully fixed. When set to low, a single treatment comparison was selected to have IPD regardless of the number of nodes. When set to medium, these increased to 2 treatment comparisons for 3-node networks, 3 for 5-node networks, and 4 for 10-node networks. When set to high, the number of treatment comparisons selected to have IPD was set to all 3 for 3-node networks, 4–6 for 5 node networks, and 5–15 for 10 node networks. Following this, a random sample of trials along these treatment comparisons were selected to have IPD. The random selection of the number of trials served to help vary the proportion of patients for which IPD was available, which is a central feature in the research question.

Having identified the number of trials and patients, and having assigned them to treatment arms, it was then possible to construct the observed outcomes. Without loss of generality, the generated data were continuous rather than dichotomous, count or otherwise; principally for computing speed. The mean observed change in the pre-defined outcome of interest *y*_*jk*_ in the case of aggregate data and *y*_*ijk*_ in the case of IPD (a separate observation for each patient *i*)*;* and a standard error *se*_*jk*_ for the mean change in each arm for aggregate data and a standard deviation *sd*_*ijk*_ in the case of IPD.

For an IPD trial with no effect-modification, the data were generated using:

1 $$ {\displaystyle \begin{array}{l}{y}_{ijk}=\Big\{\begin{array}{ll}{\mu}_j& \kern8.22em \mathrm{if}\ {t}_k=1\\ {}{\mu}_j+{\delta}_{jk}& \kern8.2em \mathrm{if}\ {t}_k>1\end{array}\\ {}{\delta}_{jk}\sim Normal\left({d}_{t_k},\tau \right)\end{array}} $$

Where *μ*_*j*_ is the study effect that was generated using a random uniform distribution between − 3 and 6. The treatment-effects were constant from replication to replication. To be clear, d_5_ was only used in instances where the number of nodes was set to 5 or 10. The standard deviation was set to 1.0 and the heterogeneity was set 0.03, which can be considered moderate.

For an AgD trial with no effect-modification, the data were generated using:

2 $$ {\displaystyle \begin{array}{l}{y}_{jk}=\Big\{\begin{array}{llllllllllllll}{\mu}_j& & & & & & & & & & & & & if\ {t}_k=1\\ {}{\mu}_j+{\delta}_{jk}& & & & & & & & & & & & & if\ {t}_k>1\end{array}\\ {}{\delta}_{jk}\sim Normal\left({d}_{t_k},\tau \right)\end{array}} $$

Effect-modification was a result of a single covariate, *X*, that was generated in two steps. To ensure variability at the aggregate level, the aggregate values of *X*, *x*. *agg*_*j*_, were generated using a random normal distribution with a mean 0.75 and a standard deviation of 0.25. For trials that had IPD, the covariate values *x*_*ijk*_ were generated using a random uniform distribution centered at *x*. *agg*_*j*_ and extended by 0.35 in either direction. When constant effect-modification was called for, the data for IPD trials was generated in the following manner:

3 $$ {\displaystyle \begin{array}{cc}{y}_{ijk}=\Big\{\begin{array}{c}{\mu}_j+{\beta}_1{x}_{ijk}\\ {}{\mu}_j+{\beta}_1{x}_{ijk}+{\delta}_{jk}+{\beta}_2{x}_{ijk}\end{array}\operatorname{}& \begin{array}{c} if\ {t}_k=1\\ {} if\ {t}_k>1\end{array}\\ {}{\delta}_{jk}\sim Normal\left({d}_{t_k},\tau \right)\end{array}} $$

Where *β*_1_ was set to 0.5 and *β*_2_ was set to 1. The data were generated in the same way and aggregated in the case of aggregate trials. When the effect-modification was set to exchangeable, the slopes for each treatment were generated using a random normal distribution. For full transparency, the code used to generate the data is provided in the Web Appendix.

Data were generated over 200 simulations for each specific set of parameter combinations. The choice of 200 simulations was on the basis of balancing computing time and having a minimal number of simulations to not be overly influenced by a single occurrence. According to Burton et al., the choice regarding the number of simulations can be based on the accuracy of an estimate of interest, such as a regression coefficient [9]. Given that this study did not aim to estimate a specific parameter, this approach was not used. A review of simulation studies pertaining to NMAs suggests a similar distribution of number of simulations in this field, with at least one study using less than 200 simulations per scenario [10]. With 200 simulations per set of parameter permutations and a total of 108 permutations (3 node setting × 3 proportion IPD × 3 effect-modification settings × 2 trial size settings × 2 network density settings), a total of 21,600 analyses were conducted (see p.3 of Web Appendix for computer and run-time details).

### Data analysis

For each simulation, following the data generation described above, three analyses were conducted: 1) AgD-NMA; 2) AgD-NMA-MR; and 3) IPD-NMA. In all three cases, the NMA were modeled using a random-effects approach given that the data were generated using between-study heterogeneity. Specifically, the model used for AgD-NMA was:

4 $$ {\displaystyle \begin{array}{l}{\theta}_{jk}=\Big\{\begin{array}{ll}{\mu}_{jb}& \kern2.5em \mathrm{if}\ k=b\\ {}{\mu}_{jb}+{\delta}_{jb k}& \kern2.5em \mathrm{if}\ k\succ b\end{array}\\ {}{\delta}_{jb k}\sim Normal\left({d}_{bk},{\sigma}^2\right)= Normal\left({d}_{Ak}-{d}_{Ab},{\sigma}^2\right)\\ {}{d}_{AA}=0,{d}_{Ak}\sim Normal\left(0,1000\right)\end{array}} $$

Where *δ*_*jbk*_ is the trial-specific treatment-effect of *k* relative to treatment *b*. These trial-specific effects are drawn from a random-effects distribution: *δ*_*jbk*_~*N*(*d*_*bk*_, *σ*^2^). The pooled effects, *d*_*bk*_, are identified by expressing them in terms of the reference treatment A. The heterogeneity *σ*^2^ is assumed constant for all treatment comparisons.

The model used for AgD-NMA-MR was:

5 $$ {\displaystyle \begin{array}{l}{\theta}_{jk}=\Big\{\begin{array}{llll}{\mu}_{jb}& & & if\ k=b\\ {}{\mu}_{jb}+{\delta}_{jb k}& & & if\ k\succ b\end{array}\\ {}{\delta}_{jb k}=\Big\{\begin{array}{llll} Normal\left({d}_{Ak}-{d}_{Ab}+\sum \limits_l\left({\beta}_{lk}-{\beta}_{lA}\right){x}_{lj},{\sigma}^2\right)& & & if\ b=A\\ {} Normal\left({d}_{Ak}-{d}_{Ab},{\sigma}^2\right)& & & if\ b\ne A\end{array}\\ {}{d}_{AA}=0,{d}_{Ak}\sim Normal\left(0,1000\right),\kern0.5em {\beta}_{lk}={b}_l,{b}_l\sim Normal\left(0,1000\right)\end{array}} $$

Where *x*_*lj*_ is the *l*^*th*^ trial-specific covariate value. *β*_*lk*_ is the corresponding treatment-by-covariate interaction term, as suggested by the NICE DSU TSD 3 document [11].

The model used for IPD-NMA was:

6 $$ {\displaystyle \begin{array}{l}\mathrm{IPD}\\ {}{\theta}_{ijk}=\Big\{\begin{array}{lll}{\mu}_{jb}+\sum \limits_l{\beta}_{0 lj}{x}_{lij}& & if\ k=b\\ {}{\mu}_{jb}+{\delta}_{jb k}+\sum \limits_l{\beta}_{0 lj}{x}_{lij}+\sum \limits_l\left({\beta}_{1 lAk}-{\beta}_{1 lAb}\right){x}_{lij}& & if\ k\succ b\end{array}\\ {}\mathrm{AgD}\\ {}{\eta}_{jk}=\Big\{\begin{array}{lll}{\lambda}_{jb}& & if\ k=b\\ {}{\lambda}_{jb}+{\delta}_{jb k}+\sum \limits_l\left({\beta}_{1 lAk}-{\beta}_{1 lAb}\right)x. ag{g}_{lj}& & if\ k\succ b\end{array}\\ {}{\delta}_{jb k}\sim Normal\left({d}_{bk},{\sigma}^2\right)= Normal\left({d}_{Ak}-{d}_{Ab},{\sigma}^2\right)\\ {}{d}_{AA}=0,{\beta}_{1 AA}=0\kern0.75em {d}_{Ak}\sim Normal\left(0,1000\right),{\beta}_{lk}={b}_l,{b}_l\sim Normal\left(0,1000\right)\end{array}} $$

For the IPD, *β*_0*j*_ is a study-specific effect of subject-level covariate *x*_*ij*_. *β*_1*Ak*_ − *β*_1*Ab*_ reflects the interaction effects of covariate *x*_*ij*_ for treatment *k* relative to control treatment *b*. k-1 different regression coefficient *β*_1*Ak*_ will be estimated by the model. Parameters of primary interest from analyses are the pooled estimates of *d*_*Ak*_, the estimates for the heterogeneity, and treatment-by-covariate interaction effects *β*_1*Ak*_.

The parameters of the different models were estimated using a Markov Chain Monte Carlo (MCMC) method. The first 15,000 iterations were discarded as ‘burn-in’, and the inferences were based on additional 10,000 iterations using two chains. Given that there were 21,600 analyses to conduct, convergence was assessed numerically for all analyses using the multivariate potential scale reduction factor (PSRF) [12]. Values above 1.1 were seen as evidence of non-convergence. While trace plots, density plots and Gelman-Rubin-Brooks (shrink factor) plots are a better, more in-depth way of assessing convergence, it was simply not feasible to do so for the entire set of simulations [12].

### Data collection and measures of comparison

The final step to each replication was collecting the results. To assess model performance, the mean squared error (MSE) and the bias of the treatment-effects and covariate estimates were collected. Additionally, the power to detect the covariate was also collected to assess coverage (i.e., the frequency at which the 95% credible interval did not contain 0 in the estimation of *β*_1_). To assist with answering the hypothesis, the proportion of treatment comparisons with IPD and the proportion of patients with IPD was also collected.

The simulations included a varying number of parameters corresponding to treatment-effects, ranging from two to nine according to the size of the network. To simplify the quantification of the simulation results across simulation scenarios, the MSE and bias measures were calculated overall treatment parameters (i.e., the bias was calculated using d_2_ and d_3_ for 3-node networks and over d_2_ through to d_10_ in 10-node networks). Moreover, given the 108 scenarios resulting from the different factor-permutations, an average over each factor-level was used as an easier way to make sense of the results. In addition to comparing the summary statistics of the MSE, a paired t-test was used to determine whether the differences were statistically differentiable. To this end, each observed MSE pair, that is, for each parameter in each instance of the analysis, the difference between the AgD-NMA-MR analysis and the IPD-NMA were calculated and the resulting sample of differences was tested using a Wilcoxon signed-rank paired test.

All analyses were performed using R version 3.5.1 (http://www.r-project.org/) and JAGS version 4.3.

## Results

### When did IPD help?

As expected, both AgD-NMA-MR and IPD-NMA outperformed the AgD-NMA (except for scenarios with no effect modification). Therefore, comparisons are focused on IPD-NMA and AgD-NMA-MR, unless specified otherwise. The use of IPD was beneficial to the estimation process in 88 of the 108 factor permutations that were explored, was neutral in 11 factor permutations and was detrimental in 9 of the 108 scenarios. The scenarios with small, neutral and negative improvements were consistently densely populated, often large, and often with a low or medium proportion of edges with IPD. Indeed, the largest benefits to IPD were observed in small networks. Overall, the results suggest that sometimes more IPD is better than having very few and that in a larger, better-populated network too few IPD will have a negligible impact on the NMA results. With respect to the scenarios where AgD-NMA-MR had a lower median MSE than IPD-NMA, 8 of the 9 scenarios were cases with an exchangeable effect-modification. The lone exception was a scenario with no effect modification. For the numeric differences of each scenario, see the Web Appendix. Having discussed the big picture, we present more detailed results in the remainder of this section.

### Treatment-effect estimation

Across all scenarios both the IPD-NMA and the AgD-NMA-MR had distributions of bias that were centered at zero. The impact of using IPD-NMA varied greatly across scenarios, from leading to a noticeably narrower distribution of bias and more precise estimates to a more negligible improvement. Averaging over these scenarios led to density plots that suggest only a moderate improvement in validity and, at times, a large improvement in precision when using IPD-NMA.

**Fig. 3:**
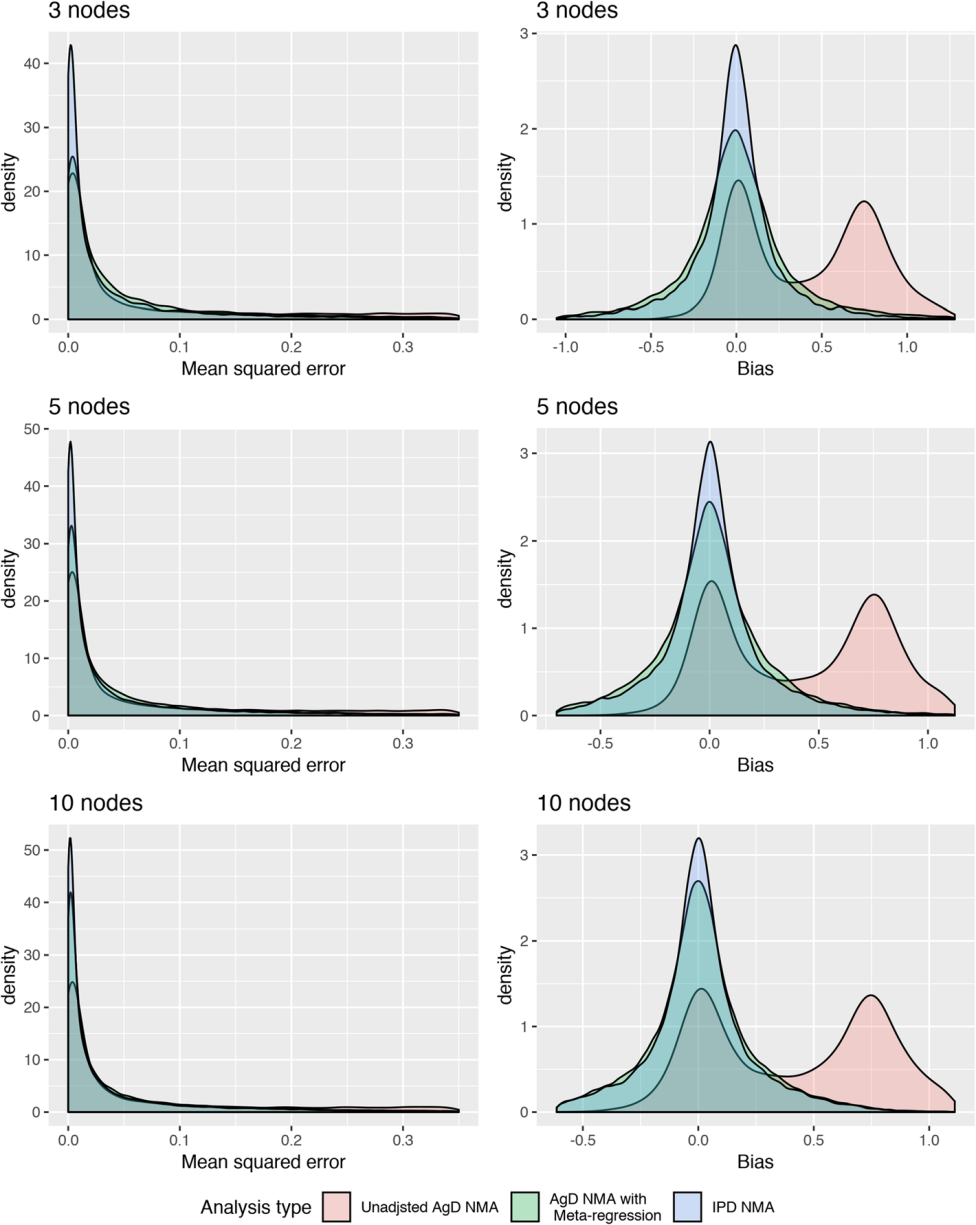
Density plots summarizing treatment-effect estimates from simulations separated by the number of nodes in the network. Legend: The mean-squared error plots on the left were limited up to 0.35 to emphasize the meta-regression analyses at the expense of undermining the mean-squared error of the unadjusted NMA

**Fig. 4:**
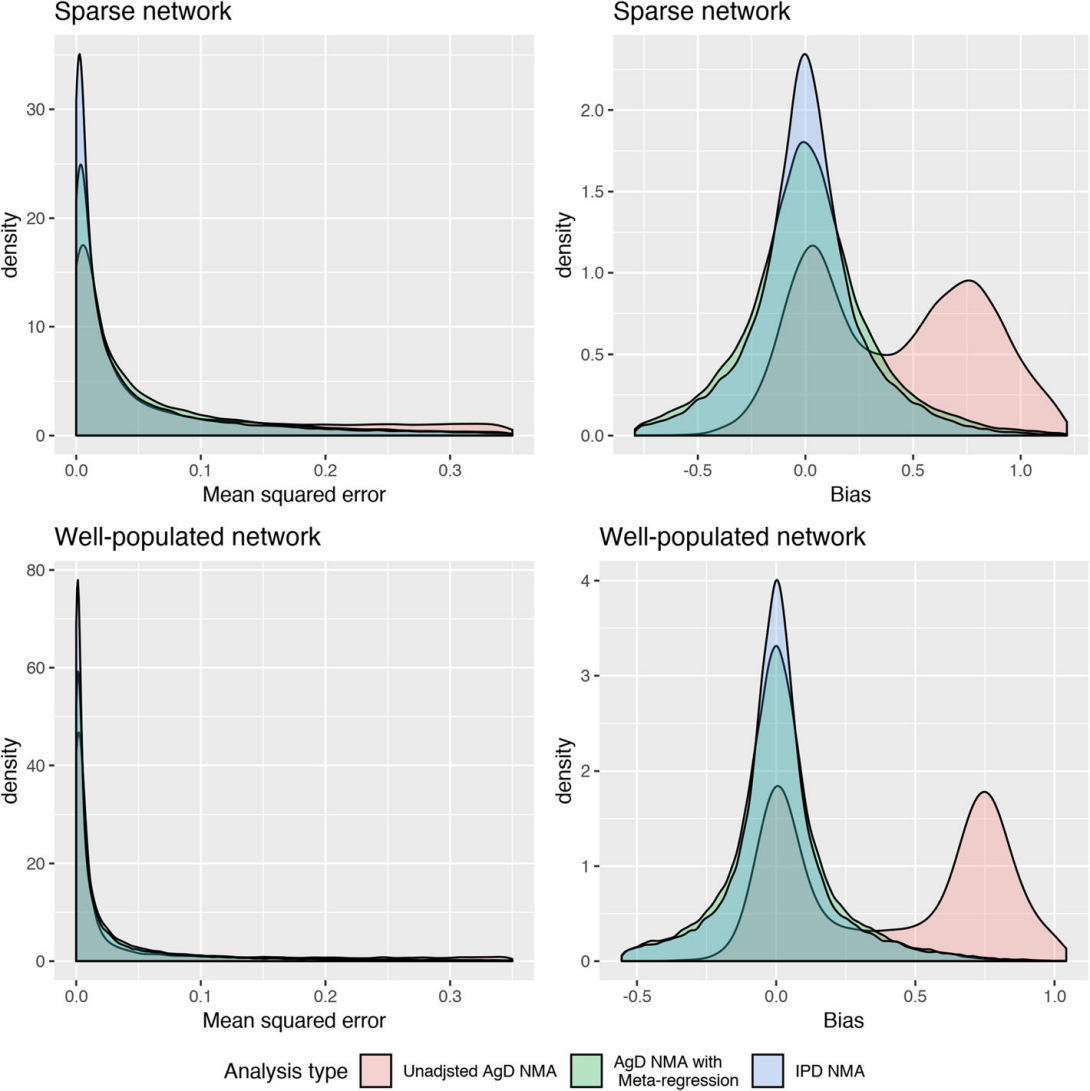
Density plots summarizing treatment-effect estimates from simulations separated by network density. Legend: The mean-squared error plots on the left were limited up to 0.35 to emphasize the meta-regression analyses at the expense of undermining the mean-squared error of the unadjusted NMA

The MSE and bias for the different numbers of nodes in the network are presented in Fig. 3. The average gained benefits of using IPD were largest in the small 3-node networks than in the larger 10 node networks. Again, this aligns with the hypothesis that the benefits of IPD may be less noticeable when there are few IPD in larger evidence networks. Similarly, as presented in Fig. 4**,** the relative difference in MSE and bias between IPD-NMA and AgD-NMA-MR was largest among sparsely populated networks. However, it should be recognized that while the difference was greatest in sparse networks, both methods performed better in the well-populated networks as these had considerably lower MSE. This was not the case with the size of network, which did not impact the MSE. Although the MSE values were small, the median MSE was 3.1 times larger for AgD-NMA-MR than in IPD-NMA in sparse networks and twice as large in well-populated networks.

**Fig. 5:**
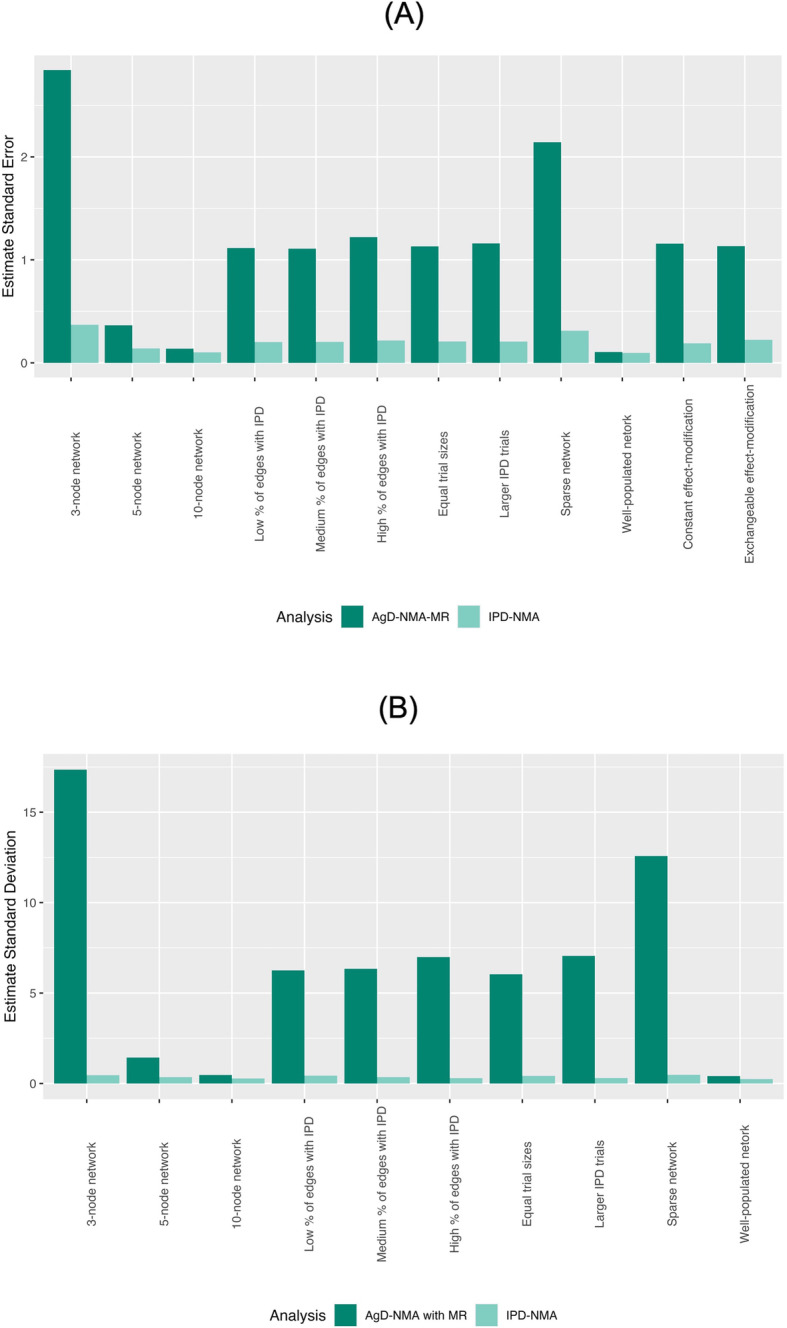
Mean standard errors using IPD-NMA and AgD-NMA-MR for (**a**) Treatment effect; and (**b**) regression coefficient

Figure 5 panel a presents the standard error of the treatment effect estimates for both the AgD-NMA-MR and the IPD-NMA averaged overall treatment-effects across each of the factor levels. Both network size and density had the largest differences across levels. On average, the benefits in terms of precision were immense in a 3-node network and negligible in a 10-node network. Similarly, for sparse and well-populated networks.

The effect of factors relating to the IPD had less impact. Neither proportion IPD nor the trial size settings had a noticeable impact on the degree of improved precision of estimation (Fig. 5a). The differences in MSE and bias between IPD-NMA and AgD-NMA-MR across the different proportions of treatment comparisons with IPD were in the expected direction (Fig. 1 *of* Web Appendix). That is to say that trials with a higher proportion of treatment comparisons with IPD had a bigger reduction in bias. Among the IPD-NMA, the median MSE went from 0.0048 to 0.0038 to 0.0026 for low, medium and high proportions of IPD, respectively, while the AgD-NMA-MR was consistent with a median MSE of 0.010 across all three scenarios. It was also reassuring that the results for the non-IPD based analyses were not affected by this factor. The difference between IPD-NMA and AgD-NMA-MR was not as pronounced for the difference between large and equal sample sizes as that observed for size and density of network (Figure 7 of the Web Appendix).

One potential issue with averaging over all scenarios is that we can lose sight of important interactions between factors. In this regard, it can be helpful to visualize differences in a factor for a specific set of factors rather than averaged over all other factors. Figures 2, 3, 4 and 5 of the Web Appendix compare the distribution of treatment-effect estimation bias in more specific scenarios. These help highlight that in larger networks the proportion of treatment comparisons with IPD matters more.

In the trivial case of having no effect-modification, the unadjusted model performed best (Figure 6 of the Web Appendix). With no effect-modification, all modeling approaches were unbiased, but the variance in the unadjusted model was considerably lower: this phenomenon was entirely expected. In the situation with effect-modification, IPD-NMA performed best given that AgD-NMA-MR had thicker tails in the bias distribution. Note that given that the bimodal behaviour of unadjusted AgD-NMA is on the basis of differences in effect-modification, by looking at each effect modification separately, the unadjusted AgD-NMA was now unimodal. Finally, the advantage of IPD-NMA relative to AgD-NMA-MR was more muted when effect-modification was exchangeable (varying from one edge to another in accordance to a Normal distribution).

### Regression coefficient estimation

Understanding how the regression coefficients are estimated can add additional insight into the results observed with respect to treatment-effect estimation. Table 2 presents the summary statistics of the MSE for the covariate coefficient estimates. For simplicity, only the simulations with a constant effect-modification were explored given that there were no covariates to estimate in simulations without effect-modification and that the estimation of MSE and bias were rendered more difficult when the covariates were generated from a random Normal distribution in the simulations with exchangeable effect-modification. The statistics regarding MSE for the regression coefficient estimates resemble those from the treatment-effect estimates. Specifically, both estimators appeared to be unbiased and the AgD-NMA-MR had a much larger range and standard deviation. As a result, the IPD-NMA had smaller MSE (both median and mean), suggesting that it leads to a reliable estimate.

**Table 2:** Summary statistics of the mean squared error of treatment-effect for the two meta-regression adjusted NMA models

Scenario	IPD Mean	IPD Median	IPD Std Dev	IPD Range	AgD MR Mean	AgD MR Median	AgD MR Std Dev	AgD MR Range
**Mean squared-error**								
Number of nodes: 3	0.014	0.004	0.035	0.811	2.065	0.015	79.472	3886.193
Number of nodes: 5	0.009	0.002	0.02	0.397	0.044	0.007	0.162	3.587
Number of nodes: 10	0.005	0.002	0.009	0.093	0.014	0.004	0.028	0.349
Proportion edges with IPD: low	0.013	0.004	0.033	0.811	1.833	0.008	79.413	3886.193
Proportion edges with IPD: medium	0.008	0.002	0.02	0.38	0.144	0.007	2.443	115.198
Proportion edges with IPD: high	0.006	0.002	0.016	0.244	0.146	0.007	2.057	91.712
Trial size: equal	0.013	0.003	0.032	0.811	0.155	0.009	2.306	115.198
Trial size: large ipd	0.006	0.002	0.013	0.38	1.26	0.006	64.853	3886.193
Network density: sparse	0.015	0.005	0.033	0.811	1.405	0.019	64.891	3886.193
Network density: well-populated	0.004	0.001	0.007	0.105	0.01	0.004	0.019	0.284
**Bias**								
Number of nodes: 3	0	0	0.119	1.517	0.029	0.005	1.437	73.842
Number of nodes: 5	0.003	0.001	0.093	1.11	0.002	0	0.209	3.773
Number of nodes: 10	0	−0.002	0.072	0.597	−0.004	−0.007	0.118	1.152
Proportion edges with IPD: low	0.003	0	0.115	1.531	0.024	0	1.354	73.842
Proportion edges with IPD: medium	0	−0.003	0.092	1.158	−0.001	−0.004	0.379	14.589
Proportion edges with IPD: high	0	0	0.079	0.977	0.003	0	0.382	13.541
Trial size: equal	0.001	−0.001	0.113	1.531	−0.006	− 0.002	0.393	18.309
Trial size: large ipd	0.001	0	0.076	0.947	0.023	0	1.123	73.842
Network density: sparse	0.002	0.001	0.121	1.531	0.017	0	1.185	73.842
Network density: well-populated	0	−0.001	0.063	0.601	0	−0.002	0.1	0.974

There was additional interest in the statistics of the regression coefficient estimates themselves because of their impact on the treatment-effect estimates. Poor estimates of the covariate coefficient will lead to poor estimates of the treatment-effects. To this end, there was a notable difference in the precision of these estimates. Figure 5 panel b presents the standard deviation of the regression coefficient estimates for both the AgD-NMA-MR and the IPD-NMA across each of the factor levels. As can be seen, the very same patterns observed in the treatment effects were observed for the regression coefficients.

### Model diagnostics

The multivariate PSRF were collected for each model (Table 4 of the Web Appendix). The summary values by analysis type are presented below in. Convergence was consistently met throughout the simulations, with the exception of very few simulations. The very small proportion of non-convergence was judged to be negligible. Scenarios where non-convergence took place were small, sparse networks and large, well-populated networks. Note that a high multivariate PSRF is not always indicative of non-convergence. Small PSRF can be obtained for each parameter and still get a large multivariate PSRF. Nonetheless, this does not happen commonly.

## Discussion

This study used simulations to explore the improvements in estimation using IPD and AgD relative to using AgD only to conduct NMA with meta-regression in accordance with numerous extrinsic and intrinsic factors of the evidence base. Study results suggest that IPD-NMA reduces estimation bias and, to a greater extent, improves the precision of treatment-effect and regression covariate estimates over NMA conducted using AgD only. On the basis of the conducted simulations, in evidence bases afflicted by effect-modifiers, the inclusion of IPD may be most impactful among small and/or sparse networks of evidence. While IPD consistently improves validity and precision in these networks, they do not always improve them in large and/or dense networks. When too few IPD are used in large or dense networks, their impact appear to be washed out and negligible. As application of IPD-NMA becomes more common in larger networks, care will be required to ensure sufficient IPD are used.

This study suggests caution in guiding users that IPD is always the approach despite the promising attributes of using IPD within NMA. The use of IPD within NMA is also quite promising. Under the strong assumptions of having access to all effect-modifiers and prognostic factors, PAIC can be used to conduct NMA with disconnected networks of evidence [13]. PAIC methods are well understood enough to warrant NICE guidance on their use [13]; however, there remain many properties of one-stage IPD-AgD NMA that remain unknown. Simulation results do help confirm and quantify some common-sense properties. Among small and/or sparsely populated networks, the use of IPD-NMA leads to significant improvements in both reduction of bias and precision of estimates. Incidentally, PAIC tends to be used in smaller networks, so use of IPD in this manner is likely to be equally impactful. Based on previous work, we hypothesized that too few IPD in large networks would lead to negligible impact – a form of washing out. Indeed, our simulations showed there needed to be at least 10% of patients in the network being from IPD in order for results to be impacted. In this way, IPD should not be included blindly, but only included in situations that could be impactful to the model estimates.

The selected circumstances were restricted to situations where it was unclear a priori whether there would be a meaningful advantage to the use of IPD-NMA. As such, all effect-modification was attributable to a single variable and the association between treatment-effect and effect-modifier was perfectly conserved at the aggregate level. For example, in the presence of ecological fallacy, the phenomenon that arises when trends in aggregate data do not match trends in individual data, using IPD will trivially be superior to AgD only [14]. There are various reasons this could happen, such as large differences in sample sizes and weights leading to Simpson’s paradox. Meta-regression in AgD-NMA is always at risk of making a model correction using a biased estimate due to the ecological fallacy and IPD is a simple way to avoid or reduce the impact of this issue. As another example, we can imagine many real-world situations where multiple effect-modifiers are imbalanced [15]. Only in exceptional circumstances can AgD be used to conduct meta-regression adjusting for multiple variables at a time. On the other hand, unless dealing with a single, small-sampled trial with IPD, IPD provides many more data points than AgD and as a result, allows for the simultaneous adjustment of multiple covariates [16]. Thus, though not demonstrated through these simulations, it is important to recognize the ability to make more complex adjustments through IPD-NMA [16]. Under both these circumstances, the added benefits of IPD are clear and there is no need to quantify these differences.

Previous studies exploring the use of IPD in combination with AgD in NMA have noted the advantages that it can bring with respect to both precision and validity. As noted by Donegan and colleagues, studies have yet to explore how much IPD is enough for the gains to be impactful [6]. A review of the literature did reveal another simulation study exploring the use of IPD and AgD for NMA [10]. Leahy and colleagues explored the benefits of IPD from the perspective of model selection, rather than bias and MSE. To this end, they found that “an increased proportion of IPD resulted in more accurate and precise estimates for most models and datasets.” They concluded that use of IPD was always beneficial relative to not having IPD. This study adds to theirs by considering the impact of size of network (theirs only considered 5-node networks), density of networks and proportion of nodes and edges available with available IPD. These studies are in agreement in that IPD is beneficial to evidence synthesis; however, our study provides further insight that too few IPD within a large network will lead to negligible benefits that may not be worth the effort.

In these simulations, the impact of IPD-NMA was more notable with respect to the increased precision of estimates. More attention was paid to the bias and MSE of the estimates; however, it is important to recognize the impact of improved precision of IPD-NMA. Improved precision leads to increased ability to correctly differentiate the impact of treatments and improve subsequent decision-making. Here too, gains were not uniform across all scenarios. Network density was the most influential factor, with improved precision most notable within sparse networks.

There are some limitations to the simulations conducted for this study. Firstly, there was no variation in the heterogeneity of studies. Network heterogeneity is an extrinsic factor that can be evaluated for a network of evidence, so understanding how the impact of IPD-NMA varies with heterogeneity would be useful to future researchers. The current study has a relatively large scale already, which led to both computational challenges and interpretational challenges, and ultimately it was not included in the study scope in order to control the complexity of the simulations. Secondly, the AgD generated for the simulations can be improved and made more realistic in future simulations, particularly when working with large sample sizes for IPD trials. By aggregating the IPD data, the residual standard error at the aggregate level was much smaller than at the individual level in some settings.

Simulation analyses represent a powerful research tool that can provide important insights into IPD-AgD NMA. While our analyses have shed light on some popular methods, future research could be expanded to much more than the suggestions that arose from our limitations above. Chief among them are simulations to expand these simulations to other IPD-AgD methods. As previously mentioned, PAIC methods tend to be restricted to small networks and the questions around large networks do not apply. Nonetheless, there are methods that have been developed to overcome the ecological fallacy in larger networks, such as those developed by Jackson et al. [14] Properties of these methods are not well understood. As such, comparisons through simulations to other IPD-AgD methods as well as the impact of the factors explored in this analysis would help shed light both on those methods as well as the differences in impact of ecological fallacy in AgD and IPD-AgD models.

## Conclusion

This study illustrates the value of IPD for network meta-analysis, but also shows that it is not a panacea. The effects of too few IPD in too large a network will get washed out in the analysis and fail to provide the potential advantages of including IPD. Nonetheless, in most circumstances, IPD can be used to improve the validity and precision of treatment-effects, which in turn leads to more useful model results.

## Supplementary Information

**Additional file 1.** When does use of individual patient data make a difference? A simulation study – Web Appendix. Description: R Code to generate data.

## Data Availability

Not applicable. Code to generate data provided in Web Appendix.

## Abbreviations

AgD: Aggregate data
AgD-NMA: Aggregate data network meta-analysis without adjustments
AgD-NMA-MR: Aggregate data network meta-analysis with meta-regression adjustments
IPD: Individual patient data
IPD-NMA: Network meta-analysis with meta-regression adjustments, using both individual patient data and aggregate data
MAIC: Matched indirect comparisons
MCMC: Markov Chain Monte Carlo
MSE: Mean squared error
NICE: National Institute for Health Care and Excellence
NMA: Network meta-analysis
PAIC: Population adjusted indirect comparisons
PSRF: Potential scale reduction factor
