# Supplementary material for: When does the use of individual patient data in network meta-analysis make a difference? A simulation study
Source: BMC Med Res Methodol. 2021 Jan 13;21:21. doi: 10.1186/s12874-020-01198-2 (PMC7805229; doi:10.1186/s12874-020-01198-2)
Supplement: Supplementary file 1 — Additional file 1. When does use of individual patient data make a difference? A simulation study – Web Appendix. Description: R Code to generate data. [file 12874_2020_1198_MOESM1_ESM.docx]

| When does use of individual patient data make a difference? A simulation study |
| --- |
| *Web Appendix* |
|  |

Contents

[Additional Methods 3](#_Toc36220568)

[Additional Results 4](#_Toc36220569)

[Simulation code 21](#_Toc36220570)

### Additional Methods

**Web-Table 1** provides further details regarding the number of trials and edges relative to the combination of network density and number of nodes.

Web-Table 1: Make of network according to density and number of nodes

| Density | 3 nodes | 5 nodes | 10 nodes |
| --- | --- | --- | --- |
| Sparse | - A 1-trial edge  - A 3-trial edge | - Two 1-trial edges  - Two 3-trial edges | - Three 1-trial edges  - Three 2-trial edges  - Three 3-trial edges |
| Well populated | - A 3-trial edge  - A 5-trial edge  - A 7-trial edge | - Two 3-trial edges  - Three 5-trial edges  - Two 7-trial edges | - Fifteen 1-trial edges  - Five 3-trial edges  - Four 7-trial edges |

* Edges are treatment comparisons

Simulations were conducted using a 2.9 GHz Intel Core i9 -8950HK CPU with 6 cores and an average of 6 parallel processes, as well as a 3.0 GHz Intel Core i5-8500B 6 cores with an average of 10 parallel processes, both of which had access to 32 GB of RAM memory. Using these resources, choosing 200 replications led to just over 3800 hours of computing. Run time varied from 8 seconds for small, sparse networks, to 75 minutes for dense, 10 node networks with large amounts of IPD.

### Additional Results

Web-Figure 1: Density plots summarizing treatment-effect estimates from simulations separated by the proportion of network edges with individual patient data


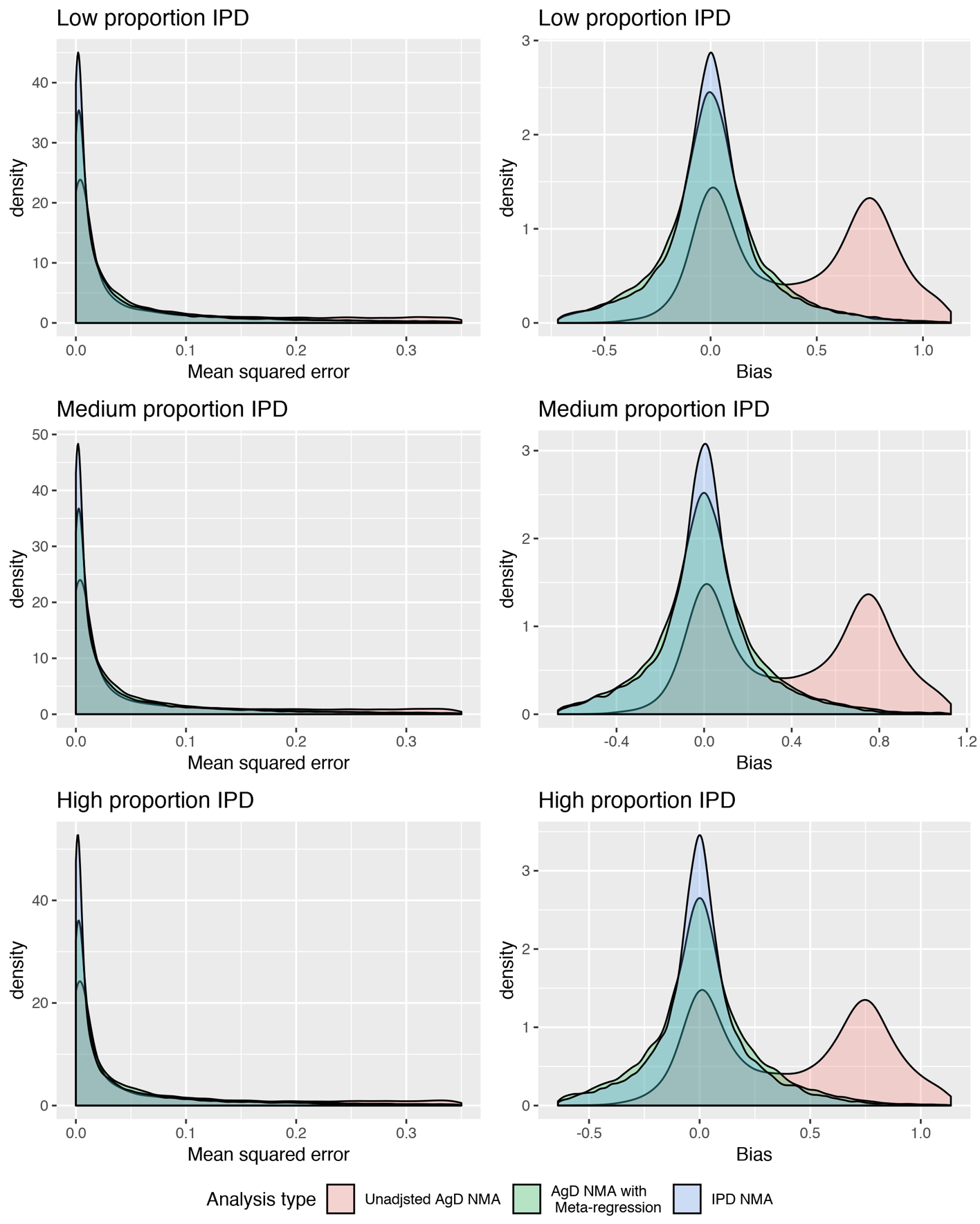


***Legend:*** *The mean-squared error plots on the left were limited up to 0.35 to emphasize the meta-regression analyses at the expense of undermining the mean-squared error of the unadjusted NMA.*

Web-Figure 2: Density of bias of treatment-effect estimates among sparse networks with 10 nodes, constant effect-modification and low proportion of edges with IPD


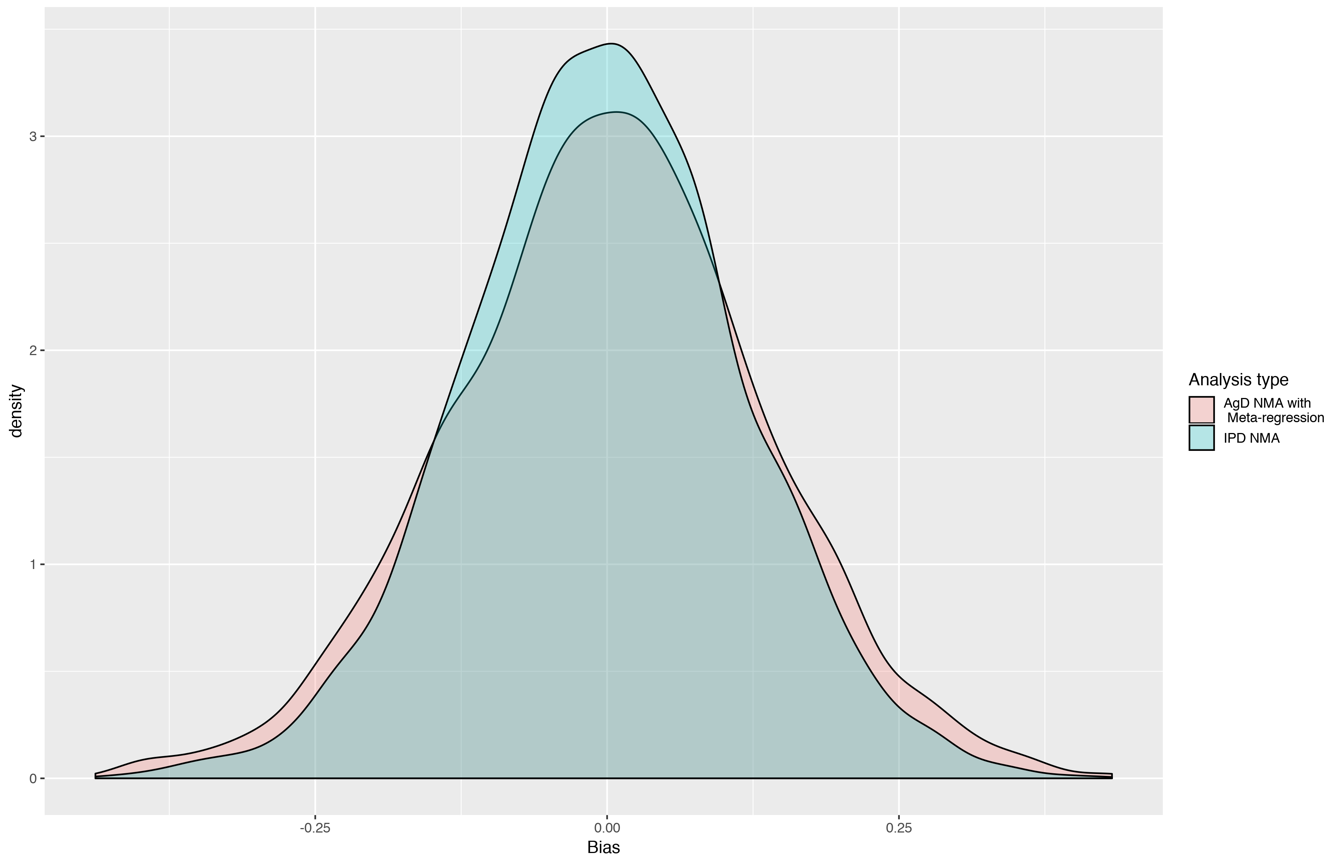


Web-Figure 3: Density of bias of treatment-effect estimates among sparse networks with 10 nodes, constant effect-modification and high proportion of edges with IPD


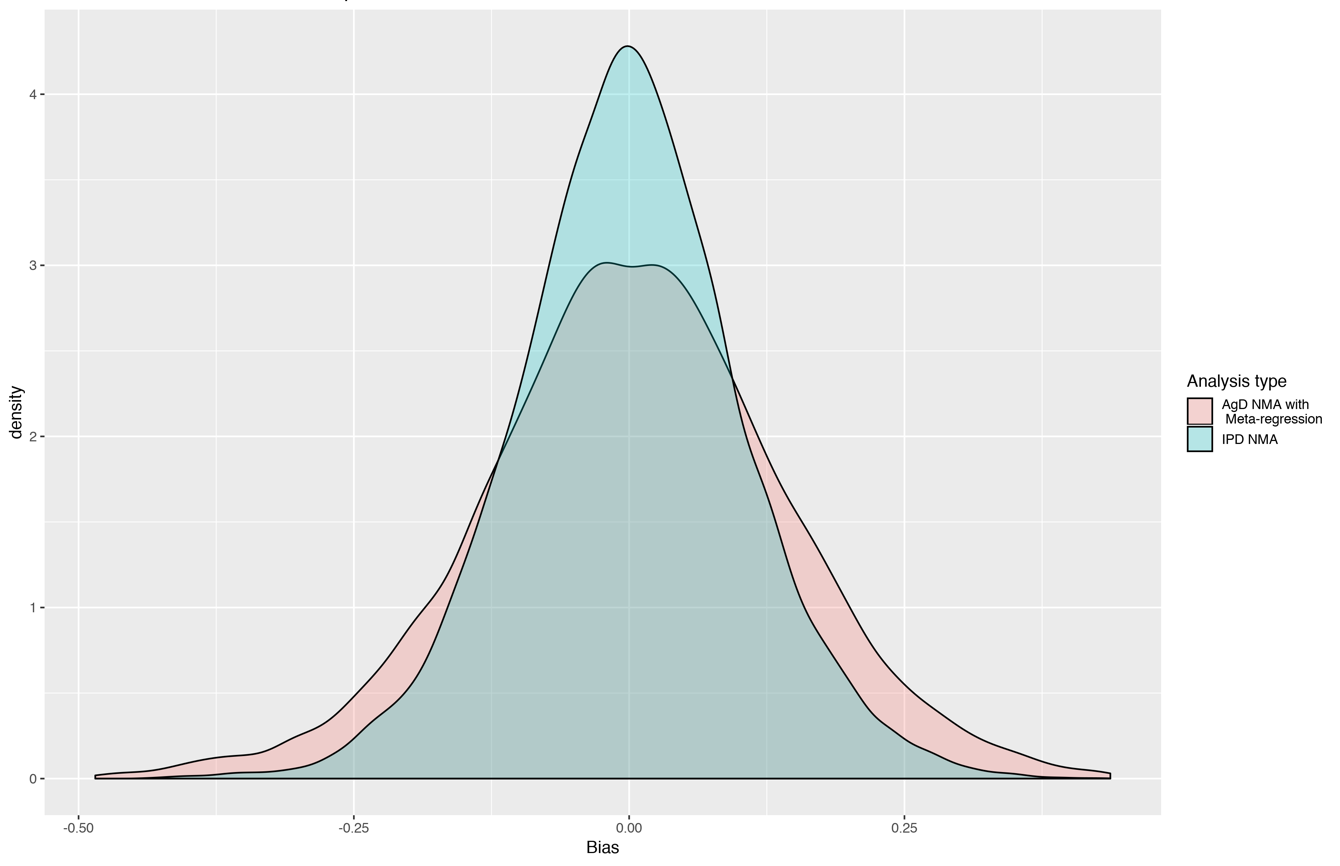


Web-Figure 4: Density of bias of treatment-effect estimates among sparse networks with 3 nodes and effect-modification


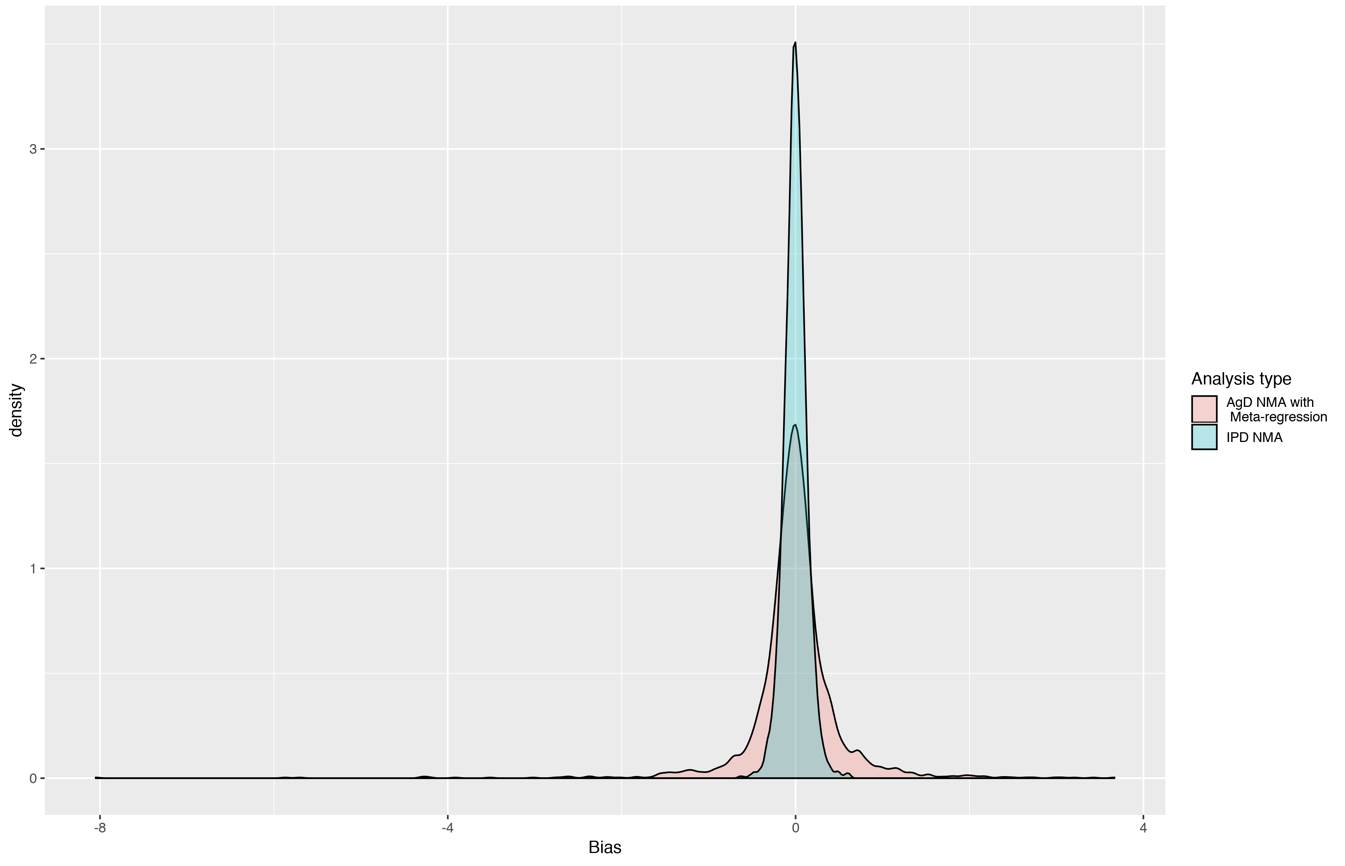


Web-Figure 5: Density of bias of treatment-effect estimates among sparse networks with 3 nodes and exchangeable effect-modification


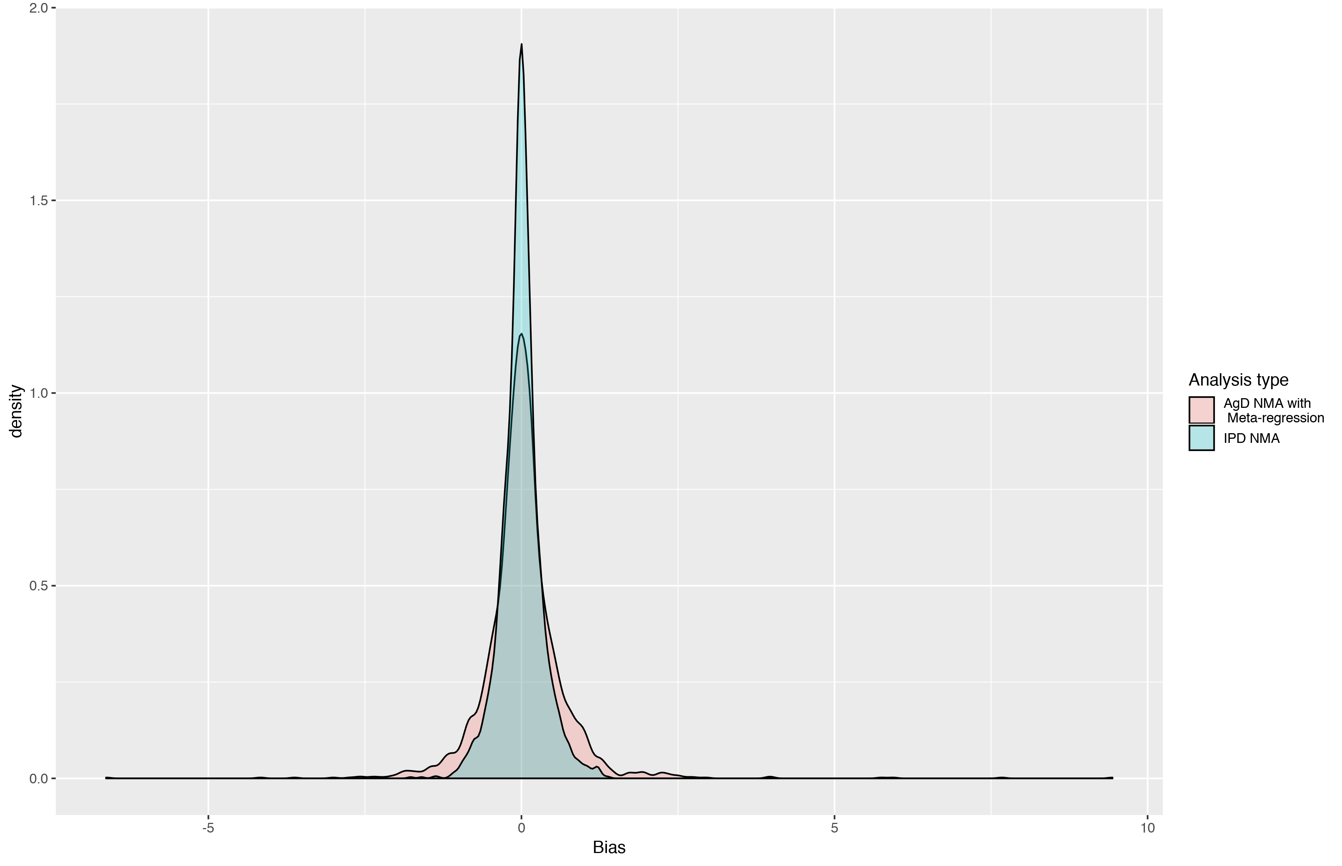


Web-Figure 6: Density plots summarizing treatment-effect estimates from simulations separated by the effect-modification


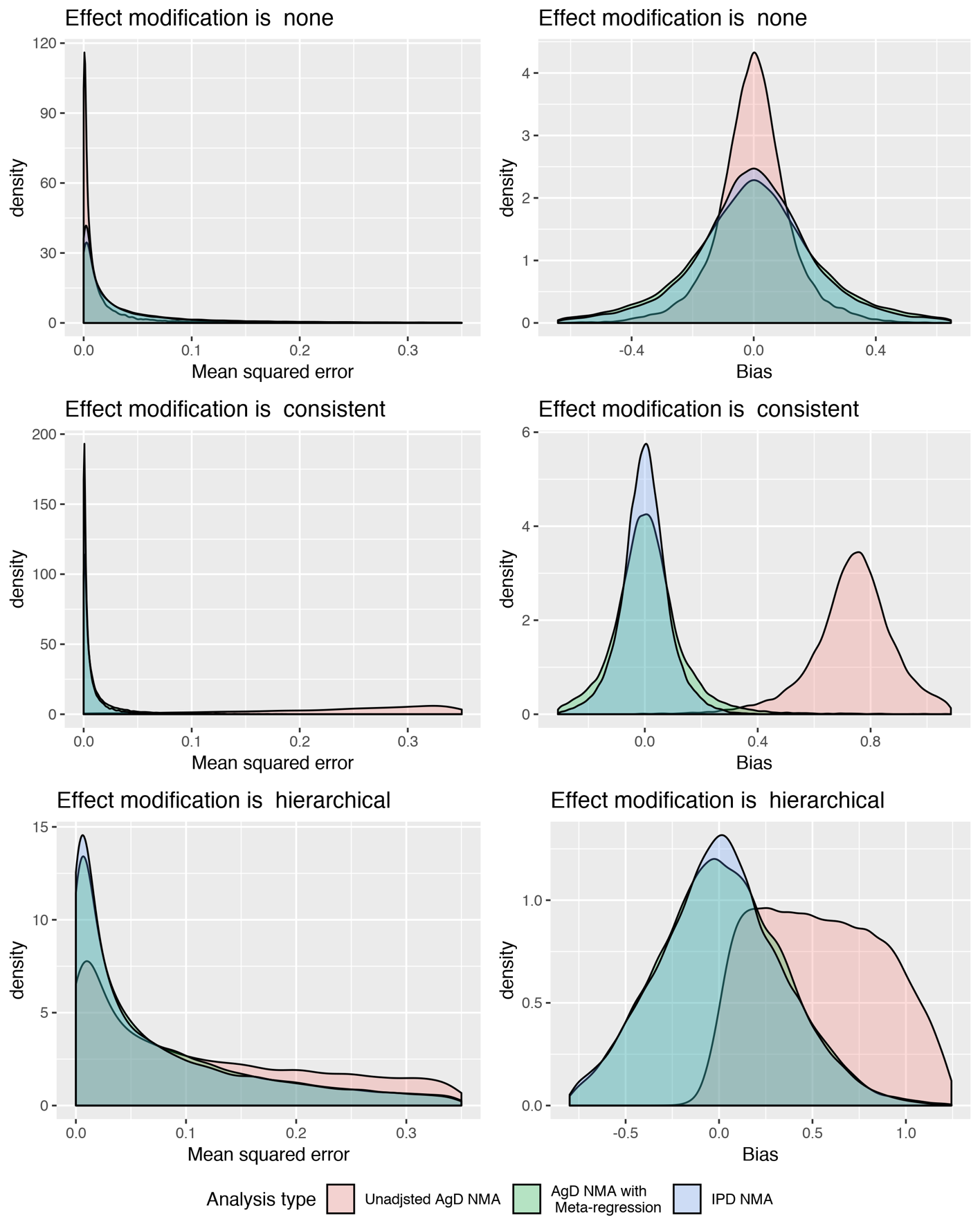


***Legend:*** *The mean-squared error plots on the left were limited up to 0.35 to emphasize the meta-regression analyses at the expense of undermining the mean-squared error of the unadjusted NMA.*

Web-Figure 7: Density plots summarizing treatment-effect estimates from simulations separated by the trial sizes among individual patient data


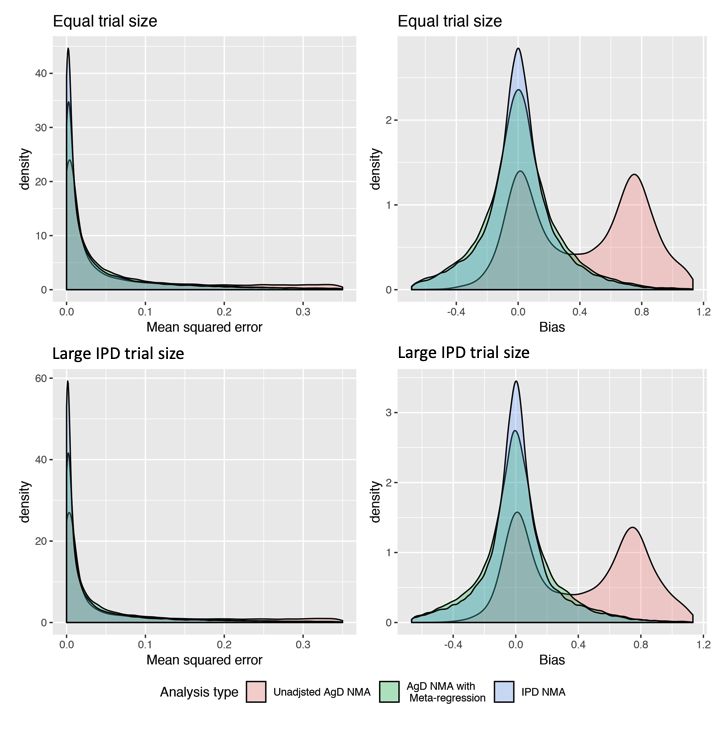


***Legend:*** *The mean-squared error plots on the left were limited up to 0.35 to emphasize the meta-regression analyses at the expense of undermining the mean-squared error of the unadjusted NMA.*

Web-Figure 8: Proportion of patients and impact on mean squared error


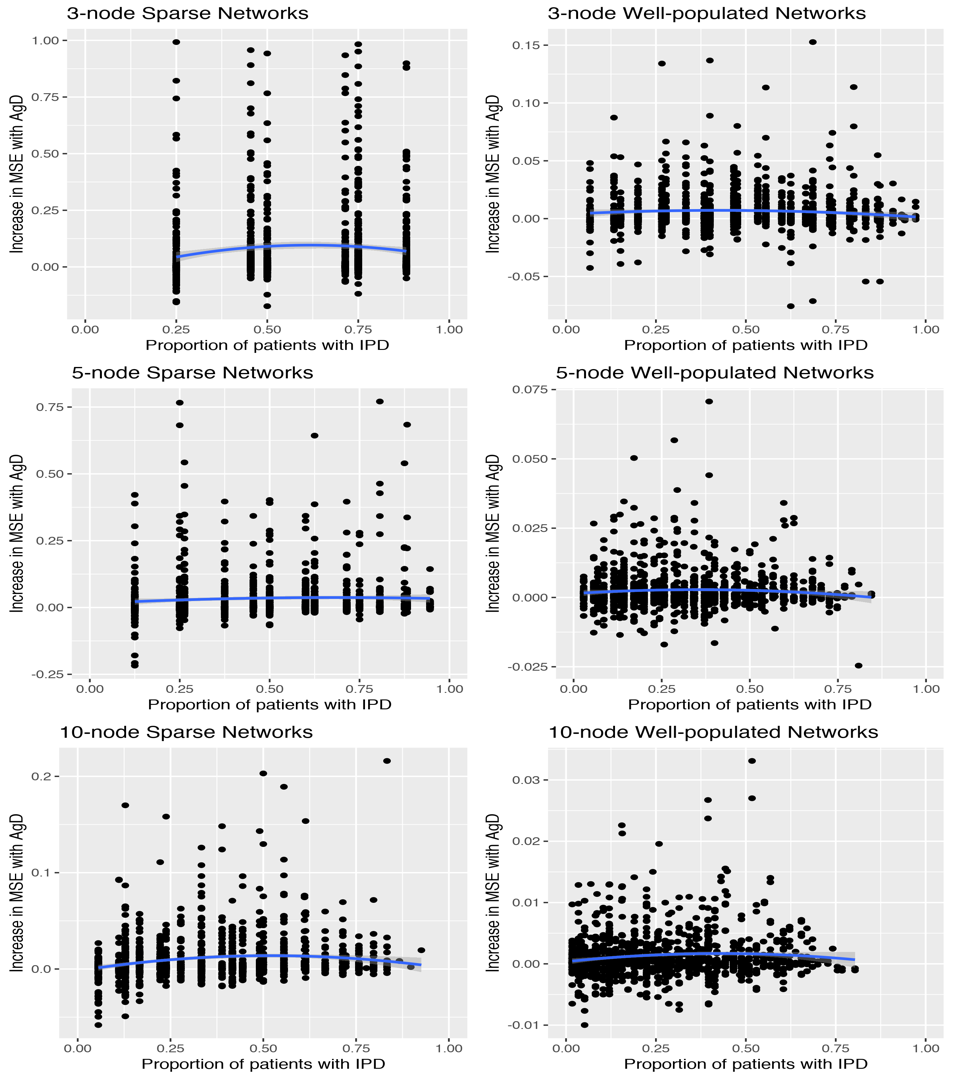


**Legend:** Each point is the difference between the average MSE using AgD-NMA-MR and IPD-NMA on the same data. Lines were obtained using loess approximation

Web-Table 2: Summary statistics of the mean squared error of treatment-effect for the two meta-regression adjusted NMA models

| Number of nodes | Proportion of edges with IPD | Effect-modification | Trial size | Density | IPD Mean | IPD Median | IPD 95^th^ percentile | IPD Maximum | AgD Meta-regression Mean | AgD Meta-regression Median | AgD Meta-regression 95^th^ percentile | AgD Meta-regression Maximum |
| --- | --- | --- | --- | --- | --- | --- | --- | --- | --- | --- | --- | --- |
| 3 | Low | Constant | Equal | Sparse | 0.031 | 0.013 | 0.032 | 0.415 | 0.459 | 0.030 | 0.144 | 34.385 |
| 3 | Medium | Constant | Equal | Sparse | 0.024 | 0.010 | 0.027 | 0.362 | 0.630 | 0.035 | 0.149 | 106.61 |
| 3 | High | Constant | Equal | Sparse | 0.018 | 0.007 | 0.019 | 0.249 | 0.288 | 0.040 | 0.132 | 9.431 |
| 3 | Low | Constant | Equal | Well-populated | 0.008 | 0.003 | 0.008 | 0.069 | 0.015 | 0.007 | 0.020 | 0.147 |
| 3 | Medium | Constant | Equal | Well-populated | 0.006 | 0.003 | 0.007 | 0.087 | 0.013 | 0.006 | 0.015 | 0.178 |
| 3 | High | Constant | Equal | Well-populated | 0.007 | 0.002 | 0.005 | 0.711 | 0.012 | 0.007 | 0.015 | 0.116 |
| 3 | Low | Constant | Large IPD | Sparse | 0.013 | 0.005 | 0.015 | 0.125 | 12.319 | 0.018 | 0.084 | 3516.68 |
| 3 | Medium | Constant | Large IPD | Sparse | 0.012 | 0.004 | 0.013 | 0.363 | 0.304 | 0.029 | 0.105 | 17.72 |
| 3 | High | Constant | Large IPD | Sparse | 0.008 | 0.003 | 0.009 | 0.119 | 0.572 | 0.022 | 0.136 | 64.811 |
| 3 | Low | Constant | Large IPD | Well-populated | 0.004 | 0.002 | 0.004 | 0.070 | 0.010 | 0.005 | 0.012 | 0.089 |
| 3 | Medium | Constant | Large IPD | Well-populated | 0.154 | 0.002 | 0.006 | 11.004 | 0.010 | 0.003 | 0.010 | 0.169 |
| 3 | High | Constant | Large IPD | Well-populated | 0.455 | 0.002 | 0.017 | 8.788 | 0.007 | 0.003 | 0.008 | 0.164 |
| 5 | Low | Constant | Equal | Sparse | 0.026 | 0.010 | 0.028 | 0.359 | 0.044 | 0.015 | 0.042 | 0.687 |
| 5 | Medium | Constant | Equal | Sparse | 0.015 | 0.007 | 0.017 | 0.198 | 0.063 | 0.016 | 0.043 | 2.913 |
| 5 | High | Constant | Equal | Sparse | 0.014 | 0.005 | 0.016 | 0.253 | 0.070 | 0.020 | 0.064 | 1.971 |
| 5 | Low | Constant | Equal | Well-populated | 0.004 | 0.002 | 0.005 | 0.045 | 0.007 | 0.003 | 0.008 | 0.069 |
| 5 | Medium | Constant | Equal | Well-populated | 0.004 | 0.002 | 0.005 | 0.046 | 0.006 | 0.003 | 0.007 | 0.058 |
| 5 | High | Constant | Equal | Well-populated | 0.003 | 0.001 | 0.004 | 0.027 | 0.006 | 0.003 | 0.008 | 0.085 |
| 5 | Low | Constant | Large IPD | Sparse | 0.014 | 0.006 | 0.017 | 0.175 | 0.065 | 0.020 | 0.054 | 4.995 |
| 5 | Medium | Constant | Large IPD | Sparse | 0.009 | 0.004 | 0.010 | 0.101 | 0.028 | 0.009 | 0.029 | 0.743 |
| 5 | High | Constant | Large IPD | Sparse | 0.006 | 0.002 | 0.006 | 0.090 | 0.048 | 0.014 | 0.044 | 1.128 |
| 5 | Low | Constant | Large IPD | Well-populated | 0.003 | 0.001 | 0.003 | 0.053 | 0.006 | 0.002 | 0.006 | 0.095 |
| 5 | Medium | Constant | Large IPD | Well-populated | 0.004 | 0.001 | 0.003 | 0.990 | 0.005 | 0.002 | 0.005 | 0.081 |
| 5 | High | Constant | Large IPD | Well-populated | 0.003 | 0.001 | 0.002 | 0.547 | 0.004 | 0.001 | 0.004 | 0.044 |
| 10 | Low | Constant | Equal | Sparse | 0.015 | 0.007 | 0.019 | 0.180 | 0.017 | 0.008 | 0.022 | 0.197 |
| 10 | Medium | Constant | Equal | Sparse | 0.011 | 0.005 | 0.014 | 0.173 | 0.022 | 0.010 | 0.026 | 0.317 |
| 10 | High | Constant | Equal | Sparse | 0.010 | 0.004 | 0.012 | 0.137 | 0.025 | 0.009 | 0.028 | 0.338 |
| 10 | Low | Constant | Equal | Well-populated | 0.004 | 0.002 | 0.005 | 0.045 | 0.005 | 0.002 | 0.006 | 0.052 |
| 10 | Medium | Constant | Equal | Well-populated | 0.003 | 0.002 | 0.004 | 0.049 | 0.005 | 0.002 | 0.006 | 0.046 |
| 10 | High | Constant | Equal | Well-populated | 0.003 | 0.001 | 0.004 | 0.047 | 0.004 | 0.002 | 0.005 | 0.075 |
| 10 | Low | Constant | Large IPD | Sparse | 0.013 | 0.005 | 0.015 | 0.186 | 0.020 | 0.007 | 0.022 | 0.363 |
| 10 | Medium | Constant | Large IPD | Sparse | 0.009 | 0.004 | 0.010 | 0.155 | 0.019 | 0.007 | 0.020 | 0.479 |
| 10 | High | Constant | Large IPD | Sparse | 0.006 | 0.002 | 0.007 | 0.091 | 0.017 | 0.006 | 0.017 | 0.383 |
| 10 | Low | Constant | Large IPD | Well-populated | 0.003 | 0.001 | 0.003 | 0.056 | 0.004 | 0.002 | 0.005 | 0.073 |
| 10 | Medium | Constant | Large IPD | Well-populated | 0.002 | 0.001 | 0.003 | 0.031 | 0.004 | 0.002 | 0.005 | 0.062 |
| 10 | High | Constant | Large IPD | Well-populated | 0.002 | 0.001 | 0.002 | 0.029 | 0.004 | 0.002 | 0.005 | 0.032 |
| 3 | Low | Exchangeable | Equal | Sparse | 0.136 | 0.028 | 0.128 | 2.544 | 1.057 | 0.056 | 0.284 | 179.795 |
| 3 | Medium | Exchangeable | Equal | Sparse | 0.123 | 0.029 | 0.099 | 1.951 | 2.995 | 0.068 | 0.312 | 782.069 |
| 3 | High | Exchangeable | Equal | Sparse | 0.087 | 0.032 | 0.094 | 1.460 | 0.535 | 0.069 | 0.317 | 35.222 |
| 3 | Low | Exchangeable | Equal | Well-populated | 0.087 | 0.018 | 0.080 | 1.226 | 0.073 | 0.028 | 0.087 | 1.046 |
| 3 | Medium | Exchangeable | Equal | Well-populated | 0.079 | 0.020 | 0.079 | 1.182 | 0.080 | 0.026 | 0.079 | 0.815 |
| 3 | High | Exchangeable | Equal | Well-populated | 0.081 | 0.022 | 0.082 | 1.246 | 0.076 | 0.029 | 0.086 | 1.365 |
| 3 | Low | Exchangeable | Large IPD | Sparse | 0.111 | 0.014 | 0.077 | 3.133 | 7.414 | 0.045 | 0.207 | 1928.032 |
| 3 | Medium | Exchangeable | Large IPD | Sparse | 0.107 | 0.019 | 0.106 | 1.433 | 0.275 | 0.060 | 0.289 | 4.475 |
| 3 | High | Exchangeable | Large IPD | Sparse | 0.076 | 0.026 | 0.086 | 0.754 | 1.126 | 0.075 | 0.297 | 144.601 |
| 3 | Low | Exchangeable | Large IPD | Well-populated | 0.100 | 0.011 | 0.102 | 1.365 | 0.075 | 0.028 | 0.079 | 1.040 |
| 3 | Medium | Exchangeable | Large IPD | Well-populated | 0.240 | 0.027 | 0.092 | 6.830 | 0.078 | 0.026 | 0.074 | 1.557 |
| 3 | High | Exchangeable | Large IPD | Well-populated | 0.465 | 0.034 | 0.188 | 9.073 | 0.071 | 0.026 | 0.078 | 0.994 |
| 5 | Low | Exchangeable | Equal | Sparse | 0.165 | 0.047 | 0.178 | 2.681 | 0.167 | 0.057 | 0.181 | 3.581 |
| 5 | Medium | Exchangeable | Equal | Sparse | 0.127 | 0.045 | 0.151 | 1.345 | 0.187 | 0.056 | 0.208 | 2.608 |
| 5 | High | Exchangeable | Equal | Sparse | 0.102 | 0.035 | 0.107 | 1.473 | 0.19 | 0.057 | 0.175 | 4.682 |
| 5 | Low | Exchangeable | Equal | Well-populated | 0.113 | 0.043 | 0.129 | 1.041 | 0.095 | 0.047 | 0.129 | 0.820 |
| 5 | Medium | Exchangeable | Equal | Well-populated | 0.112 | 0.048 | 0.131 | 1.401 | 0.094 | 0.047 | 0.109 | 0.709 |
| 5 | High | Exchangeable | Equal | Well-populated | 0.087 | 0.039 | 0.107 | 1.029 | 0.084 | 0.045 | 0.110 | 0.776 |
| 5 | Low | Exchangeable | Large IPD | Sparse | 0.169 | 0.041 | 0.188 | 2.377 | 0.187 | 0.056 | 0.169 | 5.320 |
| 5 | Medium | Exchangeable | Large IPD | Sparse | 0.121 | 0.049 | 0.142 | 1.262 | 0.167 | 0.060 | 0.197 | 2.130 |
| 5 | High | Exchangeable | Large IPD | Sparse | 0.102 | 0.041 | 0.136 | 1.377 | 0.200 | 0.056 | 0.210 | 5.209 |
| 5 | Low | Exchangeable | Large IPD | Well-populated | 0.124 | 0.029 | 0.130 | 1.113 | 0.090 | 0.041 | 0.109 | 1.017 |
| 5 | Medium | Exchangeable | Large IPD | Well-populated | 0.109 | 0.039 | 0.141 | 1.323 | 0.094 | 0.043 | 0.115 | 1.430 |
| 5 | High | Exchangeable | Large IPD | Well-populated | 0.102 | 0.042 | 0.111 | 1.945 | 0.093 | 0.042 | 0.114 | 0.874 |
| 10 | Low | Exchangeable | Equal | Sparse | 0.150 | 0.064 | 0.179 | 2.523 | 0.142 | 0.058 | 0.169 | 2.035 |
| 10 | Medium | Exchangeable | Equal | Sparse | 0.129 | 0.058 | 0.160 | 2.539 | 0.141 | 0.059 | 0.170 | 2.627 |
| 10 | High | Exchangeable | Equal | Sparse | 0.118 | 0.058 | 0.145 | 2.170 | 0.149 | 0.064 | 0.166 | 2.319 |
| 10 | Low | Exchangeable | Equal | Well-populated | 0.118 | 0.060 | 0.149 | 1.310 | 0.111 | 0.060 | 0.140 | 1.059 |
| 10 | Medium | Exchangeable | Equal | Well-populated | 0.118 | 0.066 | 0.149 | 1.535 | 0.110 | 0.063 | 0.145 | 1.219 |
| 10 | High | Exchangeable | Equal | Well-populated | 0.101 | 0.054 | 0.133 | 0.836 | 0.106 | 0.054 | 0.138 | 1.038 |
| 10 | Low | Exchangeable | Large IPD | Sparse | 0.183 | 0.065 | 0.22 | 2.289 | 0.143 | 0.062 | 0.173 | 2.141 |
| 10 | Medium | Exchangeable | Large IPD | Sparse | 0.130 | 0.054 | 0.157 | 2.726 | 0.143 | 0.062 | 0.169 | 2.656 |
| 10 | High | Exchangeable | Large IPD | Sparse | 0.117 | 0.056 | 0.148 | 1.940 | 0.144 | 0.060 | 0.168 | 2.932 |
| 10 | Low | Exchangeable | Large IPD | Well-populated | 0.139 | 0.064 | 0.178 | 1.124 | 0.108 | 0.066 | 0.144 | 0.833 |
| 10 | Medium | Exchangeable | Large IPD | Well-populated | 0.118 | 0.060 | 0.146 | 1.083 | 0.103 | 0.057 | 0.129 | 1.038 |
| 10 | High | Exchangeable | Large IPD | Well-populated | 0.116 | 0.063 | 0.158 | 1.035 | 0.106 | 0.057 | 0.145 | 0.958 |
| 3 | Low | None | Equal | Sparse | 0.334 | 0.100 | 0.328 | 8.664 | 0.815 | 0.158 | 0.493 | 39.495 |
| 3 | Medium | None | Equal | Sparse | 0.231 | 0.106 | 0.227 | 4.884 | 1.969 | 0.120 | 0.433 | 154.143 |
| 3 | High | None | Equal | Sparse | 0.201 | 0.077 | 0.245 | 3.378 | 1.51 | 0.148 | 0.596 | 102.513 |
| 3 | Low | None | Equal | Well-populated | 0.039 | 0.017 | 0.046 | 0.458 | 0.05 | 0.022 | 0.050 | 0.401 |
| 3 | Medium | None | Equal | Well-populated | 0.031 | 0.013 | 0.035 | 0.399 | 0.045 | 0.018 | 0.054 | 0.532 |
| 3 | High | None | Equal | Well-populated | 0.030 | 0.013 | 0.039 | 0.239 | 0.041 | 0.018 | 0.045 | 0.668 |
| 3 | Low | None | Large IPD | Sparse | 0.146 | 0.052 | 0.163 | 2.526 | 0.744 | 0.120 | 0.317 | 45.816 |
| 3 | Medium | None | Large IPD | Sparse | 0.110 | 0.045 | 0.124 | 1.214 | 1.699 | 0.105 | 0.383 | 243.352 |
| 3 | High | None | Large IPD | Sparse | 0.098 | 0.035 | 0.095 | 1.313 | 1.199 | 0.093 | 0.312 | 116.315 |
| 3 | Low | None | Large IPD | Well-populated | 0.027 | 0.013 | 0.028 | 0.296 | 0.035 | 0.013 | 0.042 | 0.412 |
| 3 | Medium | None | Large IPD | Well-populated | 0.021 | 0.008 | 0.021 | 0.263 | 0.034 | 0.012 | 0.035 | 0.530 |
| 3 | High | None | Large IPD | Well-populated | 0.015 | 0.007 | 0.020 | 0.155 | 0.034 | 0.011 | 0.036 | 0.527 |
| 5 | Low | None | Equal | Sparse | 0.157 | 0.058 | 0.181 | 3.381 | 0.160 | 0.054 | 0.163 | 2.017 |
| 5 | Medium | None | Equal | Sparse | 0.115 | 0.045 | 0.124 | 4.437 | 0.211 | 0.066 | 0.183 | 5.348 |
| 5 | High | None | Equal | Sparse | 0.093 | 0.040 | 0.103 | 1.135 | 0.244 | 0.063 | 0.169 | 15.331 |
| 5 | Low | None | Equal | Well-populated | 0.023 | 0.011 | 0.028 | 0.209 | 0.026 | 0.012 | 0.033 | 0.245 |
| 5 | Medium | None | Equal | Well-populated | 0.020 | 0.010 | 0.025 | 0.216 | 0.026 | 0.013 | 0.035 | 0.340 |
| 5 | High | None | Equal | Well-populated | 0.019 | 0.008 | 0.023 | 0.187 | 0.025 | 0.012 | 0.031 | 0.226 |
| 5 | Low | None | Large IPD | Sparse | 0.128 | 0.046 | 0.138 | 2.026 | 0.258 | 0.054 | 0.191 | 10.689 |
| 5 | Medium | None | Large IPD | Sparse | 0.064 | 0.023 | 0.071 | 1.039 | 0.195 | 0.044 | 0.171 | 3.762 |
| 5 | High | None | Large IPD | Sparse | 0.049 | 0.020 | 0.051 | 0.630 | 0.135 | 0.040 | 0.124 | 3.283 |
| 5 | Low | None | Large IPD | Well-populated | 0.019 | 0.009 | 0.021 | 0.181 | 0.022 | 0.009 | 0.025 | 0.227 |
| 5 | Medium | None | Large IPD | Well-populated | 0.011 | 0.005 | 0.013 | 0.157 | 0.016 | 0.008 | 0.019 | 0.152 |
| 5 | High | None | Large IPD | Well-populated | 0.011 | 0.005 | 0.013 | 0.127 | 0.015 | 0.006 | 0.017 | 0.186 |
| 10 | Low | None | Equal | Sparse | 0.080 | 0.029 | 0.084 | 1.166 | 0.095 | 0.032 | 0.104 | 1.559 |
| 10 | Medium | None | Equal | Sparse | 0.071 | 0.031 | 0.083 | 1.771 | 0.081 | 0.034 | 0.091 | 1.799 |
| 10 | High | None | Equal | Sparse | 0.049 | 0.019 | 0.053 | 0.698 | 0.068 | 0.028 | 0.076 | 1.200 |
| 10 | Low | None | Equal | Well-populated | 0.018 | 0.009 | 0.021 | 0.208 | 0.019 | 0.009 | 0.022 | 0.293 |
| 10 | Medium | None | Equal | Well-populated | 0.017 | 0.008 | 0.021 | 0.201 | 0.018 | 0.009 | 0.024 | 0.254 |
| 10 | High | None | Equal | Well-populated | 0.023 | 0.006 | 0.023 | 0.392 | 0.044 | 0.012 | 0.049 | 0.604 |
| 10 | Low | None | Large IPD | Sparse | 0.065 | 0.025 | 0.076 | 0.937 | 0.075 | 0.029 | 0.081 | 1.562 |
| 10 | Medium | None | Large IPD | Sparse | 0.044 | 0.018 | 0.053 | 0.549 | 0.058 | 0.023 | 0.070 | 1.454 |
| 10 | High | None | Large IPD | Sparse | 0.036 | 0.015 | 0.039 | 0.736 | 0.054 | 0.021 | 0.059 | 0.911 |
| 10 | Low | None | Large IPD | Well-populated | 0.016 | 0.007 | 0.019 | 0.222 | 0.017 | 0.007 | 0.020 | 0.228 |
| 10 | Medium | None | Large IPD | Well-populated | 0.013 | 0.006 | 0.016 | 0.242 | 0.014 | 0.006 | 0.017 | 0.300 |
| 10 | High | None | Large IPD | Well-populated | 0.013 | 0.004 | 0.012 | 0.436 | 0.021 | 0.005 | 0.018 | 0.589 |

IPD: individual patient data; AgD: Aggregate data

Web-Table 3: Summary statistics of the mean squared error of treatment-effect for the two meta-regression adjusted NMA models

| Number of nodes | Proportion of edges with IPD | Effect-modification | Trial size | Density | Mean difference between AgD and IPD | Median difference between AgD and IPD | Standard error of differences between AgD and IPD | Paired t-test p-value |
| --- | --- | --- | --- | --- | --- | --- | --- | --- |
| 3 | Medium | Constant | Equal | Sparse | 0.6055 | 0.0166 | 0.2836 | <0.0001 |
| 3 | Low | Constant | Equal | Sparse | 0.4288 | 0.0105 | 0.1218 | <0.0001 |
| 3 | High | Constant | Equal | Sparse | 0.2696 | 0.0275 | 0.0464 | <0.0001 |
| 3 | Medium | Constant | Equal | Well-populated | 0.0068 | 0.002 | 0.0008 | <0.0001 |
| 3 | Low | Constant | Equal | Well-populated | 0.0074 | 0.0025 | 0.001 | <0.0001 |
| 3 | High | Constant | Equal | Well-populated | 0.0055 | 0.0033 | 0.002 | 0.0333 |
| 3 | Medium | Constant | Large IPD | Sparse | 0.2917 | 0.0233 | 0.0678 | <0.0001 |
| 3 | Low | Constant | Large IPD | Sparse | 12.3055 | 0.0132 | 9.1127 | <0.0001 |
| 3 | High | Constant | Large IPD | Sparse | 0.5642 | 0.0174 | 0.1967 | <0.0001 |
| 3 | Medium | Constant | Large IPD | Well-populated | 0.1437 | 0.0008 | 0.0419 | <0.0001 |
| 3 | Low | Constant | Large IPD | Well-populated | 0.0062 | 0.0024 | 0.0007 | <0.0001 |
| 3 | High | Constant | Large IPD | Well-populated | 0.4476 | 0.0002 | 0.0622 | <0.0001 |
| 5 | Medium | Constant | Equal | Sparse | 0.0474 | 0.0047 | 0.0069 | <0.0001 |
| 5 | Low | Constant | Equal | Sparse | 0.0178 | 0.0017 | 0.0029 | <0.0001 |
| 5 | High | Constant | Equal | Sparse | 0.0563 | 0.0094 | 0.0057 | <0.0001 |
| 5 | Medium | Constant | Equal | Well-populated | 0.0016 | 0.0006 | 0.0003 | <0.0001 |
| 5 | Low | Constant | Equal | Well-populated | 0.0028 | 0.0007 | 0.0003 | 0.0129 |
| 5 | High | Constant | Equal | Well-populated | 0.0031 | 0.001 | 0.0003 | 0.0124 |
| 5 | Medium | Constant | Large IPD | Sparse | 0.0193 | 0.0036 | 0.0021 | <0.0001 |
| 5 | Low | Constant | Large IPD | Sparse | 0.0513 | 0.0099 | 0.0086 | <0.0001 |
| 5 | High | Constant | Large IPD | Sparse | 0.0422 | 0.0088 | 0.0039 | <0.0001 |
| 5 | Medium | Constant | Large IPD | Well-populated | 0.0005 | 0.0005 | 0.0014 | 6e-04 |
| 5 | Low | Constant | Large IPD | Well-populated | 0.0024 | 0.0006 | 0.0003 | 0.0161 |
| 5 | High | Constant | Large IPD | Well-populated | 0.0002 | 0.0004 | 0.001 | 0.5841 |
| 10 | Medium | Constant | Equal | Sparse | 0.0111 | 0.0034 | 0.0007 | <0.0001 |
| 10 | Low | Constant | Equal | Sparse | 0.0021 | 0.0005 | 0.0005 | <0.0001 |
| 10 | High | Constant | Equal | Sparse | 0.0156 | 0.0037 | 0.0009 | <0.0001 |
| 10 | Medium | Constant | Equal | Well-populated | 0.0013 | 0.0003 | 0.0001 | <0.0001 |
| 10 | Low | Constant | Equal | Well-populated | 0.0008 | 0.0002 | 0.0001 | 0.0071 |
| 10 | High | Constant | Equal | Well-populated | 0.0013 | 0.0002 | 0.0001 | 0.0294 |
| 10 | Medium | Constant | Large IPD | Sparse | 0.01 | 0.0019 | 0.0007 | 0.0159 |
| 10 | Low | Constant | Large IPD | Sparse | 0.0077 | 0.0013 | 0.0007 | 1e-04 |
| 10 | High | Constant | Large IPD | Sparse | 0.0108 | 0.0023 | 0.0007 | 1e-04 |
| 10 | Medium | Constant | Large IPD | Well-populated | 0.0016 | 0.0007 | 0.0001 | <0.0001 |
| 10 | Low | Constant | Large IPD | Well-populated | 0.0009 | 0.0001 | 0.0001 | <0.0001 |
| 10 | High | Constant | Large IPD | Well-populated | 0.0014 | 0.0006 | 0.0001 | 0.0335 |
| 3 | Medium | Exchangeable | Equal | Sparse | 2.8714 | 0.0281 | 2.0617 | <0.0001 |
| 3 | Low | Exchangeable | Equal | Sparse | 0.9208 | 0.0199 | 0.5146 | <0.0001 |
| 3 | High | Exchangeable | Equal | Sparse | 0.448 | 0.0335 | 0.1097 | <0.0001 |
| 3 | Medium | Exchangeable | Equal | Well-populated | 0.0012 | 0.0006 | 0.0063 | 3e-04 |
| 3 | Low | Exchangeable | Equal | Well-populated | 0.0138 | 0.0006 | 0.007 | <0.0001 |
| 3 | High | Exchangeable | Equal | Well-populated | 0.005 | 0.0002 | 0.0076 | 0.4965 |
| 3 | Medium | Exchangeable | Large IPD | Sparse | 0.1677 | 0.0225 | 0.0262 | <0.0001 |
| 3 | Low | Exchangeable | Large IPD | Sparse | 7.3032 | 0.0138 | 5.0119 | <0.0001 |
| 3 | High | Exchangeable | Large IPD | Sparse | 1.0499 | 0.0333 | 0.4565 | <0.0001 |
| 3 | Medium | Exchangeable | Large IPD | Well-populated | -0.1615 | 0 | 0.0396 | 7e-04 |
| 3 | Low | Exchangeable | Large IPD | Well-populated | 0.0251 | 0.0009 | 0.0082 | <0.0001 |
| 3 | High | Exchangeable | Large IPD | Well-populated | -0.3939 | -0.0061 | 0.0635 | <0.0001 |
| 5 | Medium | Exchangeable | Equal | Sparse | 0.0593 | 0.006 | 0.01 | <0.0001 |
| 5 | Low | Exchangeable | Equal | Sparse | 0.0017 | 0.0012 | 0.0105 | <0.0001 |
| 5 | High | Exchangeable | Equal | Sparse | 0.0884 | 0.0189 | 0.0117 | <0.0001 |
| 5 | Medium | Exchangeable | Equal | Well-populated | -0.0183 | -0.0021 | 0.0051 | <0.0001 |
| 5 | Low | Exchangeable | Equal | Well-populated | -0.0184 | -0.0003 | 0.0048 | <0.0001 |
| 5 | High | Exchangeable | Equal | Well-populated | -0.0028 | 0.0003 | 0.0029 | 0.826 |
| 5 | Medium | Exchangeable | Large IPD | Sparse | 0.046 | 0.0054 | 0.0073 | <0.0001 |
| 5 | Low | Exchangeable | Large IPD | Sparse | 0.0176 | 0.0034 | 0.0107 | 0.5484 |
| 5 | High | Exchangeable | Large IPD | Sparse | 0.0974 | 0.0104 | 0.0126 | 2e-04 |
| 5 | Medium | Exchangeable | Large IPD | Well-populated | -0.0147 | -0.0005 | 0.0043 | 2e-04 |
| 5 | Low | Exchangeable | Large IPD | Well-populated | -0.0339 | -0.0012 | 0.0058 | <0.0001 |
| 5 | High | Exchangeable | Large IPD | Well-populated | 0.0088 | 0.0002 | 0.0048 | 0.0156 |
| 10 | Medium | Exchangeable | Equal | Sparse | 0.0118 | 0.0014 | 0.0041 | <0.0001 |
| 10 | Low | Exchangeable | Equal | Sparse | 0.0078 | 0.0022 | 0.0047 | <0.0001 |
| 10 | High | Exchangeable | Equal | Sparse | 0.0315 | 0.0054 | 0.0038 | <0.0001 |
| 10 | Medium | Exchangeable | Equal | Well-populated | -0.0076 | -0.0004 | 0.0023 | 1e-04 |
| 10 | Low | Exchangeable | Equal | Well-populated | -0.0069 | -0.0007 | 0.0021 | <0.0001 |
| 10 | High | Exchangeable | Equal | Well-populated | 0.0046 | 0.0008 | 0.002 | 0.2565 |
| 10 | Medium | Exchangeable | Large IPD | Sparse | 0.013 | 0.0011 | 0.0042 | <0.0001 |
| 10 | Low | Exchangeable | Large IPD | Sparse | -0.04 | -0.0041 | 0.0063 | 0.2349 |
| 10 | High | Exchangeable | Large IPD | Sparse | 0.0276 | 0.0032 | 0.004 | 7e-04 |
| 10 | Medium | Exchangeable | Large IPD | Well-populated | 0.0156 | 0.0013 | 0.0031 | 1e-04 |
| 10 | Low | Exchangeable | Large IPD | Well-populated | 0.0308 | 0.0036 | 0.0034 | <0.0001 |
| 10 | High | Exchangeable | Large IPD | Well-populated | 0.0099 | 0.003 | 0.0022 | <0.0001 |
| 3 | Medium | None | Equal | Sparse | 1.7374 | 0.0037 | 0.6191 | <0.0001 |
| 3 | Low | None | Equal | Sparse | 0.4814 | 0.0184 | 0.138 | <0.0001 |
| 3 | High | None | Equal | Sparse | 1.3084 | 0.0452 | 0.3764 | <0.0001 |
| 3 | Medium | None | Equal | Well-populated | 0.0138 | 0.0026 | 0.0031 | <0.0001 |
| 3 | Low | None | Equal | Well-populated | 0.0112 | 0.0022 | 0.0026 | <0.0001 |
| 3 | High | None | Equal | Well-populated | 0.0109 | 0.0017 | 0.003 | 0.5131 |
| 3 | Medium | None | Large IPD | Sparse | 1.5893 | 0.0328 | 0.761 | <0.0001 |
| 3 | Low | None | Large IPD | Sparse | 0.5984 | 0.0275 | 0.1452 | 0.8955 |
| 3 | High | None | Large IPD | Sparse | 1.1012 | 0.0427 | 0.3743 | <0.0001 |
| 3 | Medium | None | Large IPD | Well-populated | 0.0127 | 0.0029 | 0.0026 | <0.0001 |
| 3 | Low | None | Large IPD | Well-populated | 0.008 | 0.0003 | 0.0023 | <0.0001 |
| 3 | High | None | Large IPD | Well-populated | 0.0185 | 0.0029 | 0.0029 | <0.0001 |
| 5 | Medium | None | Equal | Sparse | 0.0954 | 0.0099 | 0.0187 | <0.0001 |
| 5 | Low | None | Equal | Sparse | 0.003 | 0.0003 | 0.0104 | <0.0001 |
| 5 | High | None | Equal | Sparse | 0.1512 | 0.0122 | 0.0296 | <0.0001 |
| 5 | Medium | None | Equal | Well-populated | 0.0062 | 0.0019 | 0.0009 | <0.0001 |
| 5 | Low | None | Equal | Well-populated | 0.0033 | 0.001 | 0.0007 | 0.2848 |
| 5 | High | None | Equal | Well-populated | 0.0055 | 0.001 | 0.0009 | 0.029 |
| 5 | Medium | None | Large IPD | Sparse | 0.1316 | 0.0113 | 0.0145 | 0.1851 |
| 5 | Low | None | Large IPD | Sparse | 0.1294 | 0.004 | 0.0258 | 4e-04 |
| 5 | High | None | Large IPD | Sparse | 0.0861 | 0.0162 | 0.0098 | 0.9076 |
| 5 | Medium | None | Large IPD | Well-populated | 0.0048 | 0.0013 | 0.0007 | <0.0001 |
| 5 | Low | None | Large IPD | Well-populated | 0.0031 | 0.0005 | 0.0006 | <0.0001 |
| 5 | High | None | Large IPD | Well-populated | 0.004 | 0.0005 | 0.0007 | <0.0001 |
| 10 | Medium | None | Equal | Sparse | 0.0099 | 0.0015 | 0.0022 | <0.0001 |
| 10 | Low | None | Equal | Sparse | 0.0148 | 0.0014 | 0.002 | <0.0001 |
| 10 | High | None | Equal | Sparse | 0.0198 | 0.0033 | 0.0023 | <0.0001 |
| 10 | Medium | None | Equal | Well-populated | 0.0014 | 0.0004 | 0.0003 | <0.0001 |
| 10 | Low | None | Equal | Well-populated | 0.0015 | 0.0002 | 0.0003 | <0.0001 |
| 10 | High | None | Equal | Well-populated | 0.0214 | 0.0024 | 0.0015 | 0.0175 |
| 10 | Medium | None | Large IPD | Sparse | 0.0135 | 0.0016 | 0.002 | <0.0001 |
| 10 | Low | None | Large IPD | Sparse | 0.0104 | 0.0005 | 0.0026 | 0.0857 |
| 10 | High | None | Large IPD | Sparse | 0.0188 | 0.0025 | 0.002 | <0.0001 |
| 10 | Medium | None | Large IPD | Well-populated | 0.0006 | 0.0001 | 0.0003 | <0.0001 |
| 10 | Low | None | Large IPD | Well-populated | 0.0006 | 0 | 0.0003 | <0.0001 |
| 10 | High | None | Large IPD | Well-populated | 0.0079 | 0.0007 | 0.001 | <0.0001 |

Web-Table 4: Summary statistics for the multivariate PSRF assessing convergence of simulation models

| Analysis type | Minimum | 1^st^ Quartile | Median | 3^rd^ Quartile | Maximum | % above 1.1 |
| --- | --- | --- | --- | --- | --- | --- |
| AgD-NMA | 1.000 | 1.001 | 1.001 | 1.003 | 1.265 | 0.06% |
| AgD-NMA-MR | 1.000 | 1.004 | 1.009 | 1.018 | 2.889 | 1.63% |
| IPD-NMA | 1.000 | 1.004 | 1.007 | 1.015 | 1.422 | 0.77% |

### Simulation code

***Function to generate data according to a given scenario***

create_data = function(number_nodes, p_edges, effect_modification, trial_size, density){

## There can be up to 10 nodes, so here are the fixed parameter values

d = c(0, 9.5, 9.0, 5.0, 9.2, 7.5, 8.5, 1.7, 9.1, 7.9)

d = d[1:(number_nodes)]

tau = 0.03

sd = 1

set.seed(1128)

B = c(0.5, 1)

BB = runif(10, 0, 1.5)

BB[1] = 0

if(number_nodes == 3){

if(density == "sparse"){

ns = 4

n = matrix(200, ns, 2)

t = matrix(c(1, 1, 1, 1, 2, 2, 2, 3), ns, 2)

}

if(density == "well-populated"){

ns = 15

n = matrix(200, ns, 2)

t = matrix(c(rep(1, 12), rep(2, 10), rep(3, 8)), ns, 2)

}

}

if(number_nodes == 5){

if(density == "sparse"){

ns = 8

n = matrix(200, ns, 2)

t = matrix(c(rep(1, 8), rep(2,3), 3, 3, 3, 4, 5), ns, 2)

}

if(density == "well-populated"){

ns = 35

n = matrix(200, ns, 2)

t = matrix(c(rep(1, 24), rep(2, 18), rep(3, 7), rep(4, 5), rep(5, 5), rep(3, 5), rep(4, 3), rep(5, 3)), ns, 2)

}

}

if(number_nodes == 10){

if(density == "sparse"){

ns = 18

n = matrix(200, ns, 2)

t = matrix(c(rep(1, 18), 2, 2, 2, 3, 3, 3, 4, 4, 4, 5, 5, 6, 6, 7, 7, 8, 9, 10), ns, 2)

}

if(density == "well-populated"){

ns = 58

n = matrix(200, ns, 2)

t = matrix(c(rep(1, 43), rep(2, 8), rep(3, 7), rep(2, 7), rep(3, 7), rep(4, 7), rep(5, 7), rep(6, 3), rep(7, 3), rep(8, 3), rep(9, 3), rep(10, 3), 3, 4, 5, 6, 7, 8, 9, 10, 4, 5, 6, 7, 8, 9, 10), ns, 2)

}

}

nt = number_nodes

na = rep(2, ns)

max_n = 2

y = se = matrix(NA, ns, max_n)

nt1 = sum(t[,1] == 1)

x_agg = rnorm(ns, 0.75, 0.25)

AgD_MR = AgD_data = list(ns = ns, nt = nt, na = na, maxn = max_n, t = t)

IPD_data = list( nt = nt, na = na, maxn = max_n, nc = 1)

arm1 = arm2 =rep(0, (nt*(nt-1)/2))

count = 0

for(i in 1:(nt-1)){

arm1[(i:(nt-1))+ count] = i

arm2[(i:(nt-1))+ count] = (i+1):nt

count = count + nt-i-1

}

edge = data.frame(edge = 1:(nt*(nt-1)/2), arm1, arm2 )

if(p_edges == "low"){

## Step 1: Select the edge that will have the IPD

selected = edge[ round(runif(1, 0.5, nt-0.51), 0),]

temp = (t == as.numeric(selected[2])) + (t == as.numeric (selected[3]))

rows_ipd = (1:ns)[(rowSums(temp, na.rm = TRUE) == 2) & (na ==2)]

# Our IPD code only allows for two arms, so keep it to that

if(sum((rowSums(temp, na.rm = TRUE) == 2) & (na ==2)) == 0){

unmet = TRUE

while(unmet){

selected = edge[ round(runif(1, 0.5, nt-0.51), 0),]

temp = (t == as.numeric(selected[2])) + (t == as.numeric (selected[3]))

rows_ipd = (1:ns)[(rowSums(temp, na.rm = TRUE) == 2) & (na ==2)]

if(sum((rowSums(temp, na.rm = TRUE) == 2) & (na ==2)) > 0){

unmet = FALSE

}

}

}

n_potential = length(rows_ipd)

## Step 2: Select the number of trials that have IPD on that edge

if(n_potential > 1){

num = round(runif(1, 0.5, n_potential+0.5), 0)

rows_ipd = rows_ipd[1:num]

}

}

if(p_edges == "medium"){

## Step 1: Select the edge that will have the IPD

if(number_nodes == 3){

temp = edge[ round(runif(1, 0.5, nt+0.5), 0),]

selrows = 1:3

selrows = selrows[selrows != temp]

selected = edge[selrows, ]

}else if(number_nodes == 5){

first = sample(1:(nt-1), 2)

second = sample(edge$edge, 1)

selected = edge[unique(c(first, second)), ]

}else{

first = sample(1:(nt-1), 3)

second = sample(edge$edge, 1)

selected = edge[unique(c(first, second)), ]

}

temp = (t == as.numeric(selected[1,2])) + (t == as.numeric (selected[1,3]))

rows_ipd = (1:ns)[(rowSums(temp, na.rm = TRUE) == 2) & (na ==2)]

n_ipd = length(rows_ipd)

if(n_ipd > 1){

num = round(runif(1, 0.5, n_ipd+0.5), 0)

rows_ipd = rows_ipd[1:num]

}

for(i in 2:length(selected$edge)){

temp = (t == as.numeric(selected[i,2])) + (t == as.numeric (selected[i,3]))

temp2 = (1:ns)[(rowSums(temp, na.rm = TRUE) == 2) & (na ==2)]

if(length(temp2) > 1){

num = round(runif(1, 0.5, length(temp2)+0.5), 0)

temp2 = temp2[1:num]

}

rows_ipd = c(rows_ipd , temp2)

}

rows_ipd = unique(rows_ipd)

if(length(rows_ipd) == ns){

rows_ipd = rows_ipd[-1]

}

}

if(p_edges == "high"){

## Step 1: Select the edge that will have the IPD

if(number_nodes == 3){

selected = edge[1:3, ]

}else if(number_nodes == 5){

first = sample(1:(nt-1), 3)

second = sample(edge$edge, 3)

selected = edge[unique(c(first, second)), ]

}else{

first = sample(1:(nt-1), 5)

second = sample(edge$edge, 10)

selected = edge[unique(c(first, second)), ]

}

temp = (t == as.numeric(selected[1,2])) + (t == as.numeric (selected[1,3]))

rows_ipd = (1:ns)[(rowSums(temp, na.rm = TRUE) == 2) & (na ==2)]

n_ipd = length(rows_ipd)

if(n_ipd > 1){

num = round(runif(1, 0.5, n_ipd+0.5), 0)

rows_ipd = rows_ipd[1:num]

}

for(i in 2:length(selected$edge)){

temp = (t == as.numeric(selected[i,2])) + (t == as.numeric (selected[i,3]))

temp2 = (1:ns)[(rowSums(temp, na.rm = TRUE) == 2) & (na ==2)]

if(length(temp2) > 1){

num = round(runif(1, 0.5, length(temp2)+0.5), 0)

temp2 = temp2[1:num]

}

rows_ipd = c(rows_ipd , temp2)

}

rows_ipd = unique(rows_ipd)

if(length(rows_ipd) == ns){

rows_ipd = rows_ipd[-1]

}

}

if(trial_size == "large_ipd"){

n[rows_ipd,] = 500

}

S = refarm = treat = reference = treatment = xi = yi = NULL

x = x2= rep(NA, ns)

nref = ipd_counter = 0

if(effect_modification == "none"){

for(i in 1:ns){

if(i %in% rows_ipd){

ipd_counter = ipd_counter + 1

Ni = sum(n[i,1:na[i]])

S = c(S, rep(ipd_counter , Ni))

refarm = c(refarm, rep(t[i,1], Ni))

treati = c(rep(t[i,1], n[i,1]), rep(t[i,2], n[i,2]))

xtemp = runif(Ni, x_agg[i] - 0.7, x_agg[i] + 0.7)

xi = c(xi, xtemp)

reference = c(reference, t[i,1])

treatment = c(treatment, t[i,2])

treat = c(treat, treati)

nref = nref + n[i, 1]

mu = runif(1, -3, 6)

delta1 = 0

if(t[i,1] > 1){

mu = mu + d[t[i,1]]

}

delta2 = rnorm(1, d[t[i,2]] - d[t[i,1]], tau)

ytemp = rnorm(n[i,1], mu + delta1 , sd)

yi = c(yi, ytemp)

y[i, 1] = mean(ytemp)

se[i, 1] = sd(ytemp)/sqrt(n[i,1])

ytemp = rnorm(n[i,2], mu + delta2 , sd )

yi = c(yi, ytemp)

y[i, 2] = mean(ytemp)

se[i, 2] = sd(ytemp)/sqrt(n[i,2])

x[i] = x2[i] = mean(xtemp)

}else{

x[i] = x_agg[i]

x2[i] = x_agg[i]

s.e = rep(sd, na[i])

s.e = s.e/sqrt(n[i, 1:na[i]])

mu = runif(1, -3, 6)

delta1 = 0

if(t[i,1] > 1){

mu = mu + d[t[i,1]]

}

y[i, 1] = rnorm(1, mu + delta1, s.e)

delta2 = rnorm(1 , d[t[i,2]] - d[t[i,1]], tau)

y[i, 2] = rnorm(1, mu + delta2, s.e)

se[i, 1:na[i]] = s.e

}

}

}

if(effect_modification == "constant"){

for(i in 1:ns){

if(i %in% rows_ipd){

ipd_counter = ipd_counter + 1

Ni = sum(n[i,1:na[i]])

S = c(S, rep(ipd_counter , Ni))

refarm = c(refarm, rep(t[i,1], Ni))

treati = c(rep(t[i,1], n[i,1]), rep(t[i,2], n[i,2]))

xtemp = runif(Ni, x_agg[i] - 0.7, x_agg[i]+ 0.7)

xi = c(xi, xtemp)

reference = c(reference, t[i,1])

treatment = c(treatment, t[i,2])

treat = c(treat, treati)

if(t[i,1] == 1){

nref = nref + n[i, 1]

}

mu = runif(1, -3, 6)

delta1 = 0

if(t[i,1] > 1){

mu = mu + d[t[i,1]]

}

ytemp = rnorm(n[i,1], mu + B[1]*xtemp[1:(n[i,1])] + delta1, sd )

yi = c(yi, ytemp)

y[i, 1] = mean(ytemp)

se[i, 1] = sd(ytemp)/sqrt(n[i,1])

delta2 = rnorm(n[i,2], d[t[i,2]] - d[t[i,1]], tau)

if(t[i,1] > 1){

ytemp = rnorm(n[i,2], mu + B[1]*xtemp[(n[i,1]+1):Ni] + delta2 , sd )

}else{

ytemp = rnorm(n[i,2], mu + B[1]*xtemp[(n[i,1]+1):Ni] + delta2 + B[2]*xtemp[(n[i,1]+1):Ni], sd)

}

yi = c(yi, ytemp)

y[i, 2] = mean(ytemp)

se[i, 2] = sd(ytemp)/sqrt(n[i,2])

x2[i] = mean(xtemp)

x[i] = x2[i]

}else{

x[i] = x_agg[i]

x2[i] = x_agg[i]

s.e = sd/sqrt(n[i, 1])

mu = runif(1, -3, 6)

delta1 = 0

if(t[i,1] > 1){

mu = mu + d[t[i,1]]

}

y[i, 1] = rnorm(1, mu + delta1, s.e)

delta2 = rnorm(1 , d[t[i,2]] - d[t[i,1]], tau)

if(t[i,1] > 1){

y[i, 2] = rnorm(1, mu + delta2, s.e)

}else{

y[i, 2] = rnorm(1, mu + delta2+ B[2]*x_agg[i], s.e)

}

se[i, 1:na[i]] = s.e

}

}

}

if(effect_modification == "exchangeable"){

for(i in 1:ns){

if(i %in% rows_ipd){

ipd_counter = ipd_counter + 1

Ni = sum(n[i,1:na[i]])

S = c(S, rep(ipd_counter , Ni))

refarm = c(refarm, rep(t[i,1], Ni))

treati = c(rep(t[i,1], n[i,1]), rep(t[i,2], n[i,2]))

xtemp = runif(Ni, x_agg[i] - 0.7, x_agg[i]+ 0.7)

xi = c(xi, xtemp)

B3 = BB[t[i,2]] - BB[t[i,1]]

reference = c(reference, t[i,1])

treatment = c(treatment, t[i,2])

treat = c(treat, treati)

if(t[i,1] == 1){

nref = nref + n[i, 1]

}

mu = runif(1, -3, 6)

delta1 = 0

if(t[i,1] > 1){

mu = mu + d[t[i,1]]

}

ytemp = rnorm(n[i,1], mu + B[1]*xtemp[1:(n[i,1])] + delta1, sd )

yi = c(yi, ytemp)

y[i, 1] = mean(ytemp)

se[i, 1] = sd(ytemp)/sqrt(n[i,1])

delta2 = rnorm(n[i,2], d[t[i,2]] - d[t[i,1]], tau)

ytemp = rnorm(n[i,2], mu + B[1]*xtemp[(n[i,1]+1):Ni] + delta2 + B3*xtemp[(n[i,1]+1):Ni] , sd )

yi = c(yi, ytemp)

y[i, 2] = mean(ytemp)

se[i, 2] = sd(ytemp)/sqrt(n[i,2])

x2[i] = mean(xtemp)

x[i] = x2[i]

}else{

B3 = BB[t[i,2]] - BB[t[i,1]]

x[i] = x_agg[i]

x2[i] = x_agg[i]

s.e = sd/sqrt(n[i, 1])

mu = runif(1, -3, 6)

delta1 = 0

if(t[i,1] > 1){

mu = mu + d[t[i,1]]

}

y[i, 1] = rnorm(1, mu + delta1, s.e[1])

delta2 = rnorm(1 , d[t[i,2]] - d[t[i,1]], tau)

y[i, 2] = rnorm(1, mu + delta2+ B3*x_agg[i], s.e)

se[i, 1:na[i]] = s.e

}

}

}

## Before loading up the list, we re-order some of them

ord = 1-(treat == refarm)

yi = yi[order(ord)]

xi = xi[order(ord)]

xi = matrix(xi, nrow = length(xi), ncol = 1)

S = S[order(ord)]

refarm = refarm[order(ord)]

treat = treat[order(ord)]

## Load up the IPD object

IPD_data$nobs = length(S)

IPD_data$nref = nref

IPD_data$ns_ipd = ipd_counter

IPD_data$ns_ag = ns - ipd_counter

IPD_data$S = S

IPD_data$refarm = refarm

IPD_data$treat = treat

IPD_data$treatment = treatment

IPD_data$reference = reference

IPD_data$nc = 1

IPD_data$xi = xi

IPD_data$yi = yi

IPD_data$x = matrix(x[-rows_ipd], nrow = length(x[-rows_ipd]), ncol = 1)

IPD_data$t = t[-rows_ipd,]

IPD_data$y = y[-rows_ipd,]

IPD_data$na = na[-rows_ipd]

IPD_data$se = se[-rows_ipd,]

if(class(IPD_data$y) == "numeric"){

IPD_data$y = matrix(IPD_data$y, nrow = 1)

}

if(class(IPD_data$t) == "numeric"){

IPD_data$t = matrix(IPD_data$t, nrow = 1)

}

if(class(IPD_data$se) == "numeric"){

IPD_data$se = matrix(IPD_data$se, nrow = 1)

}

## Load up the AgD objects

AgD_data$y = y

AgD_data$se = se

AgD_MR$y = y

AgD_MR$se = se

AgD_MR$x = matrix(x2, nrow = length(x2), ncol = 1)

AgD_MR$nc = 1

params = list(d = d, tau = tau, B = B)

prop_edge = ipd_counter/ns

prop_patients = IPD_data$nobs/sum(n, na.rm = TRUE)

return(list(IPD_data = IPD_data, AgD_MR = AgD_MR, AgD_data = AgD_data, prop_edge = prop_edge, prop_patients = prop_patients, true_params = params))

}
